# Evolutionary and Comparative Analysis of Bacterial Nonhomologous End Joining Repair

**DOI:** 10.1093/gbe/evaa223

**Published:** 2020-10-20

**Authors:** Mohak Sharda, Anjana Badrinarayanan, Aswin Sai Narain Seshasayee

**Affiliations:** e1 National Centre for Biological Sciences, Tata Institute of Fundamental Research, Bangalore, Karnataka, India; e2 School of Life Science, The University of Trans-Disciplinary Health Sciences & Technology (TDU), Bangalore, Karnataka, India

**Keywords:** double-strand breaks, phylogeny, nonhomologous end joining DNA repair, bioinformatics, bacteria, comparative genomics

## Abstract

DNA double-strand breaks (DSBs) are a threat to genome stability. In all domains of life, DSBs are faithfully fixed via homologous recombination. Recombination requires the presence of an uncut copy of duplex DNA which is used as a template for repair. Alternatively, in the absence of a template, cells utilize error-prone nonhomologous end joining (NHEJ). Although ubiquitously found in eukaryotes, NHEJ is not universally present in bacteria. It is unclear as to why many prokaryotes lack this pathway. Toward understanding what could have led to the current distribution of bacterial NHEJ, we carried out comparative genomics and phylogenetic analysis across ∼6,000 genomes. Our results show that this pathway is sporadically distributed across the phylogeny. Ancestral reconstruction further suggests that NHEJ was absent in the eubacterial ancestor and can be acquired via specific routes. Integrating NHEJ occurrence data for archaea, we also find evidence for extensive horizontal exchange of NHEJ genes between the two kingdoms as well as across bacterial clades. The pattern of occurrence in bacteria is consistent with correlated evolution of NHEJ with key genome characteristics of genome size and growth rate; NHEJ presence is associated with large genome sizes and/or slow growth rates, with the former being the dominant correlate. Given the central role these traits play in determining the ability to carry out recombination, it is possible that the evolutionary history of bacterial NHEJ may have been shaped by requirement for efficient DSB repair.

Significance

Unlike recombination-based repair, nonhomologous end joining (NHEJ) repair is ubiquitously present in eukaryotes but sporadically in prokaryotes. Outstanding fundamental questions that remain to be answered in the field include 1) what evolutionary trajectory shaped the bacterial NHEJ distribution and 2) what evolutionary forces are responsible for it. Our study shows multiple independent gain and loss events of NHEJ, resulting in a sporadic distribution across the bacterial domain, with evidence of multiple horizontal gene transfers. We also highlight the association of NHEJ with three central genome characteristics: G–C content, genome size, and growth rate. Approaches used in our study could be extended to other repair pathways in order to understand how they might have contributed to bacterial evolution or vice versa.

## Introduction

Accurate transmission of genetic material from parent to progeny is essential for the continuity of life. However, low rates of error during replication and DNA break-inducing mutagenic agents (such as ionizing radiation and reactive oxygen), while generating diversity for natural selection to act on (Tenaillon et al. 2004), also adversely affect viability and could lead to diseases including cancer (Tomasetti et al. 2017; Srinivas et al. 2019). Therefore, most cellular life forms invest in mechanisms that repair damaged DNA including double-strand breaks (DSBs).

Two major mechanisms of repair of DNA DSBs are homologous recombination and nonhomologous end joining (NHEJ). Recombination-based repair requires a homologous copy of the DNA around the damage site for repair to occur. In contrast, NHEJ, the subject of the present work, directly ligates the DSB after detecting and binding the break ends (Aniukwu et al. 2008; Bhattarai et al. 2014). Where direct ligation is not possible—at breaks that generate complex ends—a processing step involving the removal of damaged bases and resynthesis of lost DNA is required. Such processing can be error-prone (Bétermier et al. 2014). Thus, NHEJ can be a double-edged sword: required for essential DNA repair when a homologous DNA copy is not available, but also prone to causing errors at complex DNA breaks.

NHEJ is a major mechanism of DNA repair in eukaryotes. In bacteria however, homologous recombination-based repair is the most common mechanism of DNA repair; NHEJ, on the other hand, was described only recently (Aravind and Koonin 2001; Doherty et al. 2001), and its prevalence still remains to be systematically elucidated. Unlike eukaryotes, where NHEJ activity is regulated in a cell-cycle-dependent manner, it is unclear as to when NHEJ may be a preferred mode of bacterial DSB repair. Recent reports have shown that NHEJ can contribute to mutagenesis during a specific stage of bacterial growth, such as in stationary phase (Bhattarai et al. 2014; Paris et al. 2015), raising the possibility that availability of a second copy of the genome for repair and/or growth phase may dictate whether recombination or NHEJ is employed for repair.

Experiments in *Mycobacterium* and *Pseudomonas* have shown that bacterial NHEJ repair machinery consists primarily of two proteins. The homodimeric Ku binds DNA break ends and recruits the three-domain LigD harboring phosphoesterase (PE), polymerase (POL), and ligase (LIG) activity. These three domains are respectively required to process the ends, add bases if necessary, and subsequently ligate the break. Additionally, the POL domain mediates interaction between Ku and LigD (Della et al. 2004; Stephanou et al. 2007; Aniukwu et al. 2008; Zhu and Shuman 2010). As an exception, studies in *Bacillus subtilis* found that these bacteria encode Ku along with a two-domain (LIG and POL) LigD (Moeller et al. 2007). In the absence of LigD, it is also possible for LigC, which contains only the LIG domain, to carry out repair (Aniukwu et al. 2008; Bhattarai et al. 2014; Matthews and Simmons 2014). Though *Escherichia coli* does not encode NHEJ, expression of *Mycobacterium tuberculosis* Ku and LigD renders *E. coli* NHEJ proficient (Malyarchuk et al. 2007).

Studies in the early 2000s, with a small number of genomes, suggested that the distribution of NHEJ in bacteria could be patchy (Bowater and Doherty 2006). Collectively, what does it mean for bacteria like *E. coli* to not code for NHEJ and others like *Mycobacterium tuberculosis* to harbor it? Given the large population sizes and relatively short generation times that make selection particularly strong, the question of the pressures that determine the deployment of the potentially risky NHEJ assumes importance. In this study, using bioinformatic sequence searches of Ku and LigD domains in the genomic sequences of ∼6,000 bacteria, we have tried to 1) understand how pervasive their pattern of occurrence is, 2) trace their evolutionary history, and 3) understand what selection pressures could explain it.

## Materials and Methods

### Data

All “complete” and “latest” (assembly_summary.txt; as of January 2017) genome information files for ∼6,000 bacteria were downloaded from the NCBI ftp website using in-house scripts—whole genome sequences (.fna), protein coding nucleotide sequences (.fna), RNA sequences (.fna), and protein sequences (.faa). All the organisms were assigned respective phylum and subphylum based on the KEGG classification (https://www.genome.jp/kegg/genome.html; as of May 2018).

### Identification of NHEJ Repair Proteins

Initial BlastP (Altschul et al. 1990) was run using each of the four protein domain sequences—Ku and LigD (LigD-LIG, LigD-POL, and LigD-PE) from *Pseudomonas aeruginosa PAO1* as the query sequence against the UniProtKB database (UniProt Consortium 2019) with an *E*-value cutoff of 0.0001. The top 250 full-length sequence hits were downloaded from UniProt for both Ku and LigD domains, respectively. A domain multiple sequence alignment (MSA) was made using phmmer -A option with the top 250 hits as the sequence database and *P. aeruginosa* domain sequences as the query. An hmm profile was built using the hmmbuild command for the MSA obtained in the previous step. To find domain homologs, hmmsearch command with an *E*-value cutoff of 0.0001 was used with the hmm profile as the query against a database of 5,973 bacterial genome sequences (supplementary table 1, Supplementary Material online). These homolog searches were done using HMMER package v3.3 (Finn et al. 2011). An in-house python script was written to extract the results and assign organisms with Ku and LigD domains. The same methods were used to identify NHEJ components in 243 archaeal genomes (supplementary table 2, Supplementary Material online).

**Fig. 1:**
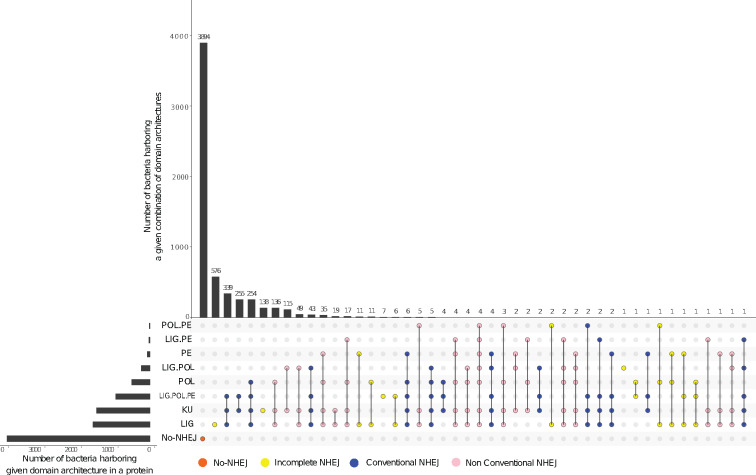
Distribution of NHEJ components in bacteria. An UpSet plot depicting the number of bacteria harboring a certain type of domain architecture per protein and number of bacteria harboring a combination of domain architecture in their respective genomes is shown for 5,973 analyzed genomes. *NHEJ−* (orange), *Ku only* (yellow), *LigD only* (yellow), *Conventional NHEJ+* (blue), and *nonconventional NHEJ+* (pink).

Bacteria were assigned to five broad categories, based on the status of NHEJ components—*NHEJ−*, *Ku only*, *LigD only*, *conventional NHEJ+*, and *nonconventional NHEJ+*. Conventional NHEJ was said to be present in bacteria harboring Ku and LigD having LIG, POL, and PE domains in the same protein. Nonconventional NHEJ included bacteria with Ku and at least one of the following: 1) all LigD domains present in different combinations in different proteins, 2) just the LIG and POL domains present in the same, or 3) different proteins, and 4) LIG with/without PE domain present in the same or different proteins. Organisms with PE and/or POL but not the LIG domain detected were assigned to *NHEJ−* state if Ku domain was not detected either; a *Ku only* state if Ku domain was detected (fig. 1).

### Ku and LigD Neighborhood Analysis

In-house python scripts were written to determine the proximity of *ku* and *ligD* on the genome using the annotation files. Taking one gene as the reference, the presence of the other gene was checked within a distance of ten genes upstream or downstream inclusive of both strands. The organization of NHEJ genes fell into three categories: 1) both genes on the same strand and within the distance range; this we call as operonic NHEJ, 2) both genes on different strands and within the distance range, and 3) genes outside the distance range. This analysis was done only for *NHEJ+* organisms which code for LigD containing all the three domains as part of a single protein.

### Identification of Ribosomal Ribonucleic Acid Sequences

A 16S ribosomal ribonucleic acid (rRNA) sequence database was downloaded from the Genomic-based 16S ribosomal RNA database (GRD) website (https://metasystems.riken.jp/grd/; last accessed November 2, 2020). A MSA and a profile of the database was made using muscle v3.8.31 (Edgar 2004) and hmmbuild (Finn et al. 2011), respectively. To detect 16S rRNA homologs in our database of 5,973 bacteria, nhmmer (Wheeler and Eddy 2013) was used with an *E*-value cutoff of 0.0001 where the GRD-based 16S rRNA sequence profile was used as the query. In-house python script was written to parse the output files and select the best hits for further analysis.

### Calculation of Genome Sizes, Growth Rates, and G–C Content

In-house python scripts were written to calculate the genome sizes (GSs), growth rates (GRs), and G–C content of all bacteria in our data set (supplementary table 1, Supplementary Material online). These calculations were made after excluding the plasmid sequence information from the whole genome sequence assembly files. Previous studies have shown a significant positive correlation between a bacteria’s GR and the number of rRNA operons harbored in its genome (Klappenbach et al. 2000; Vieira-Silva and Rocha 2010; Gyorfy et al. 2015; Roller et al. 2016). Therefore, rRNA copy number was taken as a proxy for GR.

### GS Randomization Analysis

For this analysis, 920 organisms harboring conventional NHEJ were considered. The total number of coding DNA sequences was used as the proxy for GS. Because the chance of finding a gene in a larger genome is more than that in a smaller genome, 920 genes were drawn at random from a pool of all the genes coming from 5,973 organisms in our data set and the genome coding DNA sequences size (GS) from which each gene was picked was noted. For each such iteration, the median GS was calculated. This was repeated for 100 iterations and a distribution of median GS was obtained. The nonparametric Wilcoxon rank-sum test was used to compare this random distribution with the median GS of NHEJ-harboring bacteria.

### HGT Analysis

Alien Hunter v1.7 (Vernikos and Parkhill 2006) with default options was used to predict horizontally acquired regions (HARs)—based on their oligonucleotide composition—across bacterial genomes present in our data set. In-house python scripts were used to detect Ku and LigD in the predicted HARs. NHEJ was said to be acquired through horizontal gene transfer (HGT) if both Ku and LigD were present in the predicted HARs.

Phylogenetic incongruence between the archaeal–bacterial 16S rRNA-based species tree and the corresponding gene trees (for Ku, LigD-LIG domain, and RecA) was checked using the Approximately Unbiased (AU) statistical test (Shimodaira 2002). AU test was carried out with 10,000 simulations with a constrained versus unconstrained approach (explained next). The MSA for each gene was run in an unconstrained mode for the given model parameters. Then, the alignments were run in a constrained mode with respect to their respective species tree topologies. p-AU gives the probability of identifying the gene family as having evolved according to the species, that is, the evolutionary history of the gene is the same as that of the species. This test was carried out using the -au options and -zb option implemented in IQTREE.

Further, to test the strength with which the aforementioned genes could be vertically transmitted or moved among closely or distantly related bacteria, nonparametric Mantel tests were carried out. Patristic distances were calculated between bacteria in the species tree and a given gene tree, respectively. A Pearson product–moment correlation coefficient *r* was calculated between these two distance matrices consisting of bacteria shared between the two phylogenies under test. The significance of correlation was assessed via randomization test by conducting 10,000 permutations of distance matrices. The correlation coefficient *r* was recalculated for each permutation to produce the null distribution and *P* value was obtained using the one-tailed test using the R package.

RANGER-DTL 2.0 (Bansal et al. 2018) was used to predict the donors and recipients of NHEJ HGT events among and between bacterial and archaeal species. A nonpolytomous rooted 16S rRNA-based tree with archaea as an outgroup was used as a species tree. Optimal rootings for Ku and LigD-LIG domain-based bacterial–archaeal gene trees were determined using the *OptRoot* program with default options such that the duplication–transfer–loss (DTL) reconciliation cost was minimized. *Ranger-DTL* program was used to compute the optimal DTL reconciliation of a given rooted species tree—rooted gene tree pair. In case of multiple optimal reconciliations, the program inherently reports an optimal reconciliation sampled uniformly at random. Therefore, the analysis was run for 100 simulations each with a transfer cost *T* = 1, 2, or 3 (default), that is, a total of 300 simulations. The lower the transfer cost, the more the HGT events allowed during the reconciliations. *AggregateRanger* was used to compute support values for the most frequent mappings, that is, the donor species, by accounting for the variance due to multiple optimal reconciliations and alternative event cost assignments. An in-house python script was written to back trace the most frequent recipient for a given most frequent mapping for both Ku (supplementary table 3, Supplementary Material online) and LIG domain (supplementary table 4, Supplementary Material online). The results were overlaid on the respective species phylogeny using the features provided in the iTOL server (https://itol.embl.de/login.cgi; last accessed November 2, 2020).

Sequence alignments were viewed and pruned using Jalview (Waterhouse et al. 2009). Principal component analysis of Ku domain sequences was carried out based on the method by Konishi et al. (2019).

### Phylogenetic Tree Construction

For the construction of a species tree, one 16S rRNA sequence per genome was extracted into a multi-fasta file using an in-house python script. For bacteria with multiple 16S rRNA sequences, one 16S rRNA sequence was chosen such that it minimizes the number of *N*s in that sequence and has the maximum sequence length. In order to build a pruned phylogenetic tree, 970 bacteria were randomly selected such that a genus was represented exactly once for every NHEJ state. Please note that in the case of nonconventional NHEJ, all its four subcategories, as described in the previous sections, were treated separately, when including bacteria at the genus level. A MSA was built using muscle v3.8.31 (Edgar 2004) with default options. The conserved regions relevant for phylogenetic inference were extracted from the MSA using BMGE v1.12 (Criscuolo and Gribaldo 2010). After the manual detection of the alignment, one spurious sequence was removed and the MSA was built again for 969 bacteria (supplementary table 5, Supplementary Material online). Using IQTREE v1.6.5 (Nguyen et al. 2015), a maximum-likelihood (ML)-based phylogenetic tree was built with the best model chosen as SYM + R10 (LogL = −118,152.5876, BIC = 249,946.0604). ModelFinder (-m MF option) (Kalyaanamoorthy et al. 2017) was used to choose the best model for the tree construction compared against 285 other models. Branch supports were assessed using both 1,000 ultrafast bootstrap approximations (-bb 1000 -bnni option) (Hoang et al. 2018) and SH-like approximate likelihood ratio (LR) test (-alrt 1000 option) (Guindon et al. 2010).

A similar approach was used to build a phylogeny of 1,403 (supplementary table 6, Supplementary Material online) organisms, nonredundant at the species level, comprising just two NHEJ states: 1) *NHEJ−* and 2) *conventional NHEJ+*.

For the construction of bacterial–archaeal gene trees (Ku, LigD-LIG, or RecA), only bacteria harboring one Ku and one conventional LigD in their genomes were included. Because the motivation behind the construction of gene trees was to study the origin of NHEJ in bacteria, all archaea harboring either Ku or LigD-LIG domain were included in the respective phylogeny. In species with multiple copies of RecA, RecA with the highest alignment length and identity was chosen for a given genome. The species in the bacterial–archaeal 16S rRNA-based phylogeny depended on the species included in the corresponding gene tree. The approach used to build these phylogenies was similar to the one described in the previous two paragraphs.

### NHEJ Ancestral State Reconstruction Analysis

To trace the evolutionary history of NHEJ, four discrete character states were defined as follows: *Ku only*, *LigD only*, *NHEJ−*, and *NHEJ+*. States were estimated at each node using stochastic character mapping (Bollback 2006) with 1,000 simulations provided as make.simmap() method in the R package phytools v0.6-44 (Revell 2012). The phylogenetic tree was rooted using the midpoint method and polytomies were removed by assigning very small branch lengths (10^−6^) to all the branches with zero length. The prior distribution of the states was estimated at the root of the tree. Further, by default the method assumes that the transitions between different character states occur at equal rates. This might not always be true, especially with complex traits where it is supposedly easier to lose than gain such characters. Therefore, for the estimation of transition matrix *Q*, three discrete character evolution model fits were compared: Equal Rates (ER), Symmetric (SYM), and All Rates Different (ARD). This allowed for models that incorporate asymmetries in transition rates. Based on Akaike Information Criterion (AIC) weights, the ARD model (w-AIC_ARD_ =1, w-AIC_SYM_ = 0, and w-AIC_ER_ = 0) was chosen as the best fit with unequal forward and backward rates for each character state transition. Finally, *Q* was sampled 1,000 times from the posterior probability distribution of *Q* using Markov chain Monte Carlo and 1,000 stochastic maps were simulated conditioned on each sampled value of *Q*. This strategy was used to reconstruct ancestral states for both phylogenies comprising 969 and 1,403 organisms as described in the previous sections.

Ancestral states for GS, a continuous trait, were reconstructed using fastAnc() method employed in phytools v0.6-44 based on the Brownian motion model. This model was found to be the better fit model as compared with multiple rate model (Bayesian Predictive Information Criterion [BPIC]_Brownian_ < BPIC_stable_ and Proportional Scale Reduction Factor approaching <1.1 well within 1,000,000 iterations), assessed using StableTraits (Elliot and Mooers 2014). The multiple rate model allows for the incorporation of neutrality and gradualism associated with Brownian motion and also includes occasional bursts of rapid evolutionary change. Ancestral states for GR were reconstructed by converting it into a binary trait. An organism was said to be slow growing if it encoded rRNA copy numbers less than or equal to the median rRNA copy number (median = 3) and fast growing otherwise. The reconstruction was carried out using the same approach that was used for estimating NHEJ ancestral states, as described in the previous paragraph.

### NHEJ and Genome Characteristics Phylogenetic Comparative Analysis

The two genome characteristics—GS and GR—were compared across bacteria with different NHEJ states. The distributions across bacteria with different NHEJ states were first compared assuming statistical independence of bacteria, using Wilcoxon rank-sum test, wilcox.test() in R. Next, two measures of phylogenetic signal—Pagel’s *λ* (Freckleton et al. 2002) and Blomberg’s *K* (Blomberg et al. 2003)—were used for detecting the impact of shared ancestry, for GS and GR across bacteria, using the phylosig() routine in phytools R package (Revell 2012). A phylogenetic analysis of variance (ANOVA), employed in phytools R package v0.6-44 (Revell 2012), was carried out with 1,000 simulations and Holm–Bonferonni correction to control for familywise error rate, based on a method by Garland et al. (1993), to compare the genome characteristics in a phylogenetically controlled manner. Please note that GSs and rRNA copy numbers were log_10_ transformed for all the analysis.

### Correlated Evolution Analysis

Two relationships—(NHEJ repair and GS) and (NHEJ repair and GR)—were quantified using a statistical framework. To test if changes in genome characteristics occur independently of NHEJ or whether these changes more (or less) likely to occur in lineages with (or without) NHEJ, two models of evolution were considered—independent and dependent. In the independent model, both the traits were allowed to evolve separately on a phylogenetic tree, that is, noncorrelated evolution. In the dependent model, the two traits were evolved in a nonagnostic manner, that is, correlated evolution. The NHEJ repair trait had two repair character states—NHEJ− (0) and conventional NHEJ+ (1). GSs, a continuous trait, were converted into binary state as well. For this, the mean GS of organisms with 0 or 1 NHEJ repair state was computed. A “lower” state (0) was assigned if a value was less than the mean and a “higher” state (1) if the value was more. The same approach was used to convert GRs (rRNA copy number) into a binary state.

A continuous-time Markov model approach was used to investigate correlated evolution between NHEJ repair and the genome characteristics. First, the ML approach (Pagel 1994) was used to calculate log-likelihoods for the two models of evolution per trait pair: 1) NHEJ repair and GS; 2) NHEJ repair and GR. A LR statistic was calculated for both comparisons, followed by a chi-square test to assess if the dependent model was a better fit. The degrees of freedom are given by df_chi-square test_ = (*n*_rate-dependent model_ – *n*_rate-independent model_). There are eight transition rates in the dependent model across four states (00,01,10,11) and four transition rates in the independent model across two states (0,1; 0,1). Therefore, the test was run with four degrees of freedom.

The ML approach implicitly assumes that the models used for hypothesis testing are free of errors. Therefore, to make the analysis robust, the Bayesian reverse jump Markov chain Monte Carlo (RJMCMC) approach was used to calculate the marginal log-likelihoods of the independent and dependent models of evolution (Pagel and Meade 2006). This approach takes into consideration the uncertainty and minimizes the error associated in calculating the parameters used in each of our model(s), ensuring reliable interpretations. Log Bayes factor was used to assess the better fit out of the two models.

BayesTraits v3 (Pagel and Meade 2006) was used to carry out both ML- and Bayesian RJMCMC-based correlated evolution analysis as described above for both the models. ML was run using the default parameters. Bayesian RJMCMC was run for 5,050,000 iterations, sampling every 1,000th iteration with a burn-in of 50,000. For the estimation of marginal likelihood, a stepping stone sampler algorithm was used where the number of stones was set to 100 and each stone was allowed to run for 10,000 iterations.

### Phylogenetic Logistic Regression Analysis

A method developed by Ives and Garland (2010) was used to carry out the phylogenetic logistic regression analysis provided in the R package phylolm v2.6 (Tung Ho and Ané 2014) as the subroutine phyloglm() with method = logistic_IG10. NHEJ repair was the binary-dependent variable with two states: NHEJ− (0) and conventional NHEJ+ (1). The two independent continuous variables were GS and GR. Before proceeding with the analysis, these independent variables were checked for multicollinearity by calculating variance inflation factor (VIF),

$$\text{VIF}=1/\left(1-R^{2}\right).$$

Three models were tested: 1) NHEJ ∼ GS, 2) NHEJ ∼ GR, and 3) NHEJ ∼ GS + GR. The best model was chosen according to AIC scores.

All the scripts used for analysis were written in python, perl, or R. Statistical tests and data visualizations were carried out in R and iTOL server.

All the files and scripts used and generated in this study can be found at the github repository link- https://github.com/Mohak91/bacterial_nhej_repair_evolution.

## Results

### NHEJ Is Sporadically Distributed across Bacteria

To identify NHEJ machinery across bacteria, we used the reference sequences of the Ku domain, and the LIG, POL, and PE domains of LigD from *P. aeruginosa* to search ∼6,000 complete bacterial genomes for homologs (see Materials and Methods). We defined bacteria encoding Ku and the complete, three-domain version of LigD as those harboring a conventional NHEJ system. Organisms lacking the POL and/or the PE domains of LigD, and those encoding these domains in separate proteins, were defined as those carrying nonconventional NHEJ (fig. 1).

We found NHEJ in only ∼1,300 (22%) genomes studied here. There were various combinations of Ku and LigD domains across these organisms, but a large majority (920) carried conventional NHEJ. Seventy-five percent bacteria harboring conventional NHEJ coded for Ku and LigD in a 10-kb vicinity of each other, with 60% organisms carrying Ku and LigD on the same strand of the 10-kb vicinity. Most bacteria (84%) harboring NHEJ coded for a single copy of Ku, whereas the remaining coded for 2–8 Ku copies in their genomes. For example, as reported by McGovern et al. (2016) and Kobayashi et al. (2008), we identified four Ku-encoding genes in *Sinorhizobium meliloti*. About two-thirds of NHEJ positive bacteria carried multiple copies of the LIG domain, 37% carried multiple copies of the POL domain, and 8% bacteria had multiple copies of the PE domain (supplementary table 1, Supplementary Material online). We also noticed that 138 (2.3%) organisms encoded Ku and not LigD, and 619 (10.3%) only LigD and not Ku (fig. 1 and supplementary table 1, Supplementary Material online).

**Fig. 2:**
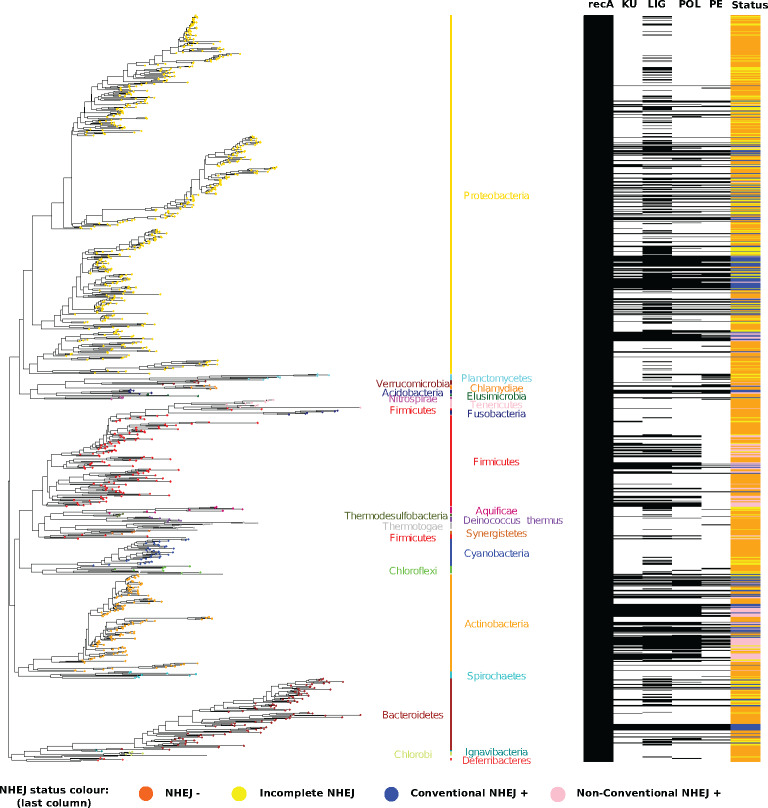
NHEJ is sporadically distributed across bacteria. 16S rRNA-based species phylogenetic tree of 969 bacterial species (left) with presence/absence matrix of RecA, KU, LIG, POL, and PE domains (right). These species were included such that each genus was chosen once for each NHEJ state (see Phylogenetic Tree Reconstruction section for further details). Tip labels, representing bacteria, are colored according to the phylum names and bars (right of the phylogenetic tree) that the given tip belongs to. The phylum names arranged on either sides of the vertical bars are for representational purposes only. The first five columns of the presence/absence matrix (extreme right of the figure) depict the status if the given protein (RecA) or domain (Ku, LIG, POL, and PE) is present (black horizontal bar) or absent (white horizontal bar) in the corresponding bacteria. Each horizontal bar maps to a bacterial tip on the phylogenetic tree (left). Horizontal bars in the last column of the matrix represent the overall NHEJ status for a given species (color legend same as in fig. 1). NHEJ status—orange: *NHEJ−*; yellow: *incomplete NHEJ*; blue: *conventional NHEJ+*; pink: *nonconventional NHEJ+*.

NHEJ was not restricted to specific bacterial classes (fig. 2) and was found in ten classes. We found a significant enrichment of conventional NHEJ in Proteobacteria (Fisher’s Exact test, *P* = 3.8 × 10^−5^, odds ratio: 2.04) and Acidobacteria (Fisher’s Exact test, *P* = 5 × 10^−2^, odds ratio: 5.286). All Bacteroidetes with NHEJ harbor a conventional NHEJ, although we could not assign statistical significance to it (Fisher’s Exact test, *P* = 0.14, odds ratio: 1.38). In contrast, nonconventional NHEJ repair was significantly overrepresented in Firmicutes (Fisher’s Exact test, *P* = 1.23 × 10^−13^, odds ratio: 6) and Actinobacteria (Fisher’s Exact test, *P* = 2.12 × 10^−15^, odds ratio: 6.14). Twenty-four phyla did not include any NHEJ positive organism (supplementary table 7, Supplementary Material online).

### NHEJ Was Gained and Lost Multiple Times through Evolution

**Fig. 3:**
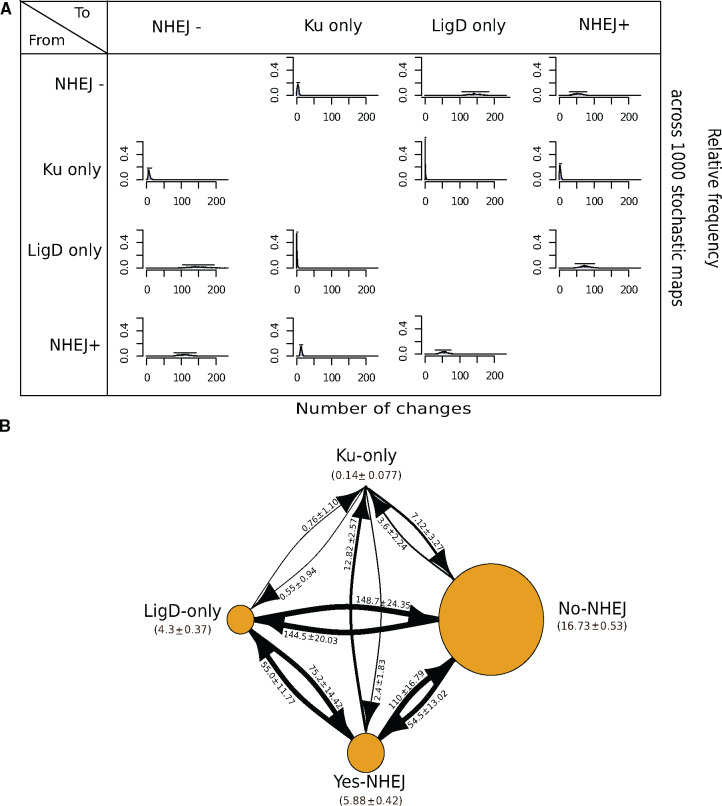
Transitions to a Ku only state are rare. (*A*) A matrix depicting relative frequency of number of changes of a state transition type across 1,000 stochastic maps. (*B*) A state transition diagram depicting the number of transitions between two given states and the time spent in each state during NHEJ evolution. The node size is proportional to the amount of time spent in a particular state. The arrow size is proportional to the number of transitions from one state to another.

We traced the number of NHEJ gains and losses starting from the eubacterial ancestor to the species at the tips of the 16S-based bacterial phylogenetic tree. To trace the evolutionary history of NHEJ, we defined four discrete character states: *Ku only*, *LigD only* (conventional and nonconventional), *NHEJ−*, and *NHEJ+*. Note that an *NHEJ+* state is defined only when both Ku and LigD are present in a bacterium. We calculated the posterior probabilities (pp) of each character state per node on the phylogeny, the distribution of the number of times each of the 12 character state transitions occurred (fig. 3*A*) and the distribution of the total time spent in each state (supplementary fig. S1, Supplementary Material online). We performed this analysis for a set of 969 genomes in which each genus was represented once for each state (see Materials and Methods).

**Fig. 4:**
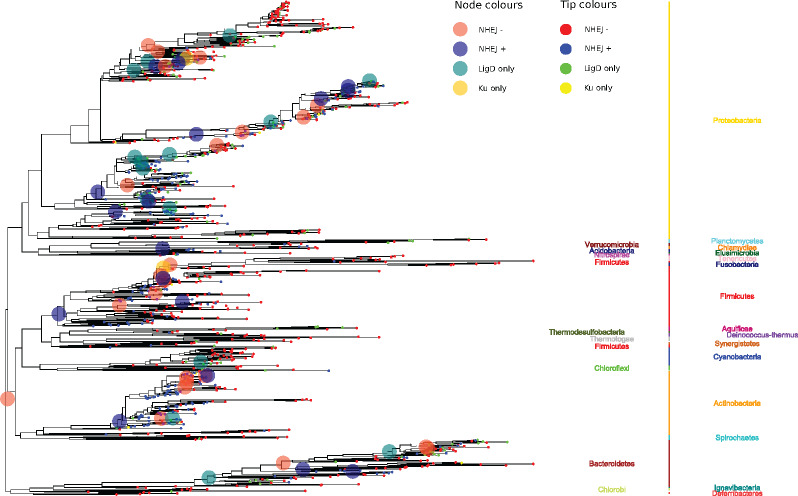
NHEJ was gained and lost multiple times through evolution. A trace of the evolutionary history of the two-component NHEJ system across 969 bacteria. These species were included such that each genus was chosen once for each NHEJ state (see Phylogenetic Tree Reconstruction section for further details). The phylum names arranged on either sides of the vertical bars are for representational purposes only. The tip and node labels are colored according to the NHEJ states—red: *NHEJ−*; yellow: *Ku only*; green: *LigD only*; blue: *NHEJ+* (conventional and nonconventional). NHEJ state for nodes is shown only when the posterior probability support is >70%; interpreted as change in NHEJ state at that node as compared with shallower phylogenetic depths.

We first asked if NHEJ was present in the common eubacterial ancestor and, given the sporadicity of NHEJ, subsequently lost in several lineages (supplementary table 8, Supplementary Material online). We assigned a major primary gain to an internal ancestral node if 1) all nodes leading to it from the root had *NHEJ−* state; 2) the pp of either *NHEJ+*, *LigD only*, or *Ku only* at that ancestral node was ≥0.7; 3) a gain of *LigD only* or *Ku only* was followed by a transition to *NHEJ+*; and 4) if it had at least three descendent species. We observed multiple major independent primary gains at ancestral nodes within Bacteroidetes, Actinobacteria, Firmicutes, Acidobacteria, and multiple subclades of Proteobacteria (supplementary table 8, Supplementary Material online). It follows that the common eubacterial ancestor likely did not have NHEJ (fig. 4).

The gain of NHEJ can be sequential, gaining either *Ku only* or *LigD only* followed by the gain of the other component; or it can be a one-step acquisition of both components (fig. 3*B*). The most common transition from an *NHEJ−* state was to a *LigD only* state. Also frequent was the direct acquisition of both components to transition from an *NHEJ−* to an *NHEJ+* state. Transition from *NHEJ−* to *Ku only* was negligible. In the reverse direction, a one-step loss of both Ku and LigD is the most likely. Again, the *Ku only* state is rare.

A one-step transition from *NHEJ−* to *NHEJ+* is likely through HGT. Sixty bacterial genomes belonging to the phyla Alpha-proteobacteria (in particular the Rhizobiales)—and Beta-proteobacteria, and Streptomycetales carried their NHEJ components on plasmids (supplementary table 1, Supplementary Material online). However, based on abnormal word usage statistics (see Materials and Methods), we could not find NHEJ to be a part of the horizontally acquired component of the chromosomes of any bacterial genome. At least two *NHEJ−* to *NHEJ+* transitions occurred close to the root, and it is possible that the predictions of horizontally acquired NHEJ systems made so far may be an underestimate (fig. 4). We investigate this in greater detail below.

In summary, 1) the common eubacterial ancestor was devoid of NHEJ; 2) NHEJ was gained and lost multiple times; and 3) transitions to a *Ku only* state are rare.

### NHEJ and HGT

Experimental studies in the archaea *Methanocella paludicola* (Bartlett et al. 2013, 2016; Brissett et al. 2013) have confirmed the presence of a functional NHEJ repair, with crystal structures revealing close relationship with the bacterial proteins (Bartlett et al. 2016; White and Allers 2018).

To assess the possibility of horizontal transfer of NHEJ machinery across prokaryotes, we first performed a domain wise search in 243 archaea to complement the data we had assembled for bacteria. These searches revealed the presence of both Ku and LigD-LIG domains in ten archaeal species (see Materials and Methods; supplementary table 2, Supplementary Material online). However, 230 archaeal genomes encoded LigD but no Ku. This lends support to previous reports suggesting that NHEJ is rare in archaea (Bartlett et al. 2013, 2016; White and Allers 2018).

**Fig. 5:**
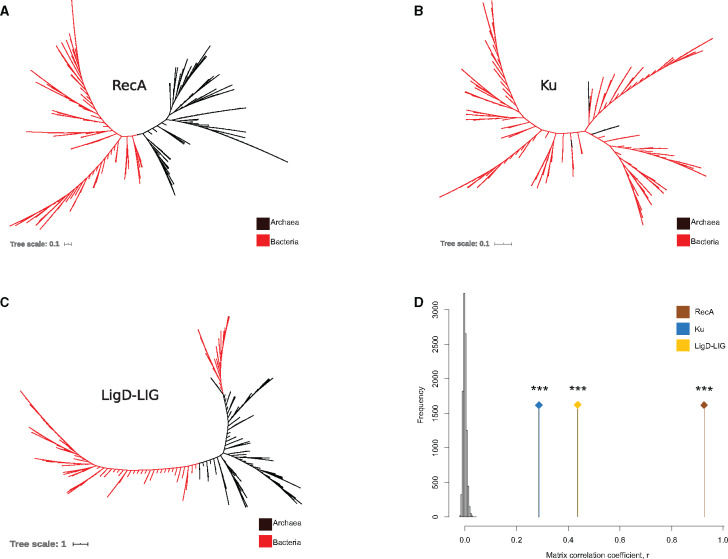
Phylogenetic methods suggest a strong role of HGT in NHEJ evolution. (*A*) Unrooted RecA tree, (*B*) unrooted Ku tree, (*C*) unrooted LigD-LIG tree, and (*D*) Mantel test correlation coefficient (*r*) comparing RecA, Ku, and LigD-LIG distance matrices with 16S rRNA distance matrices, respectively, compared against a null distribution of *r* obtained by 10,000 matrix randomizations.

In order to check for horizontal transfer events between bacteria and archaea, we used phylogenetic methods based on detecting conflicts between an organismal phylogeny and a phylogeny inferred for Ku and LigD-LIG domains, respectively. This method allowed us to test for any ancient transfers across bacteria as well. We found that NHEJ proteins undergo HGT events at significantly high rate (p-AU = 0; fig. 5*B* and *C*; see Materials and Methods) and these are not limited to closely related species or cospeciation events (fig. 5*D*; Mantel test, *P* < 10^−4^, *r*_Ku_ = 0.25; *P* < 10^−4^, *r*_LIG_ = 0.4). We also noted incongruence with respect to the RecA phylogeny (p-AU = 0; fig. 5*A*) as has been reported before (Eisen 1995; Lang et al. 2013). However, these were limited at best to transfers among closely related species (fig. 5*D*; Mantel test, *P* < 10^−4^, *r*_RecA_ = 0.94).

**Fig. 6:**
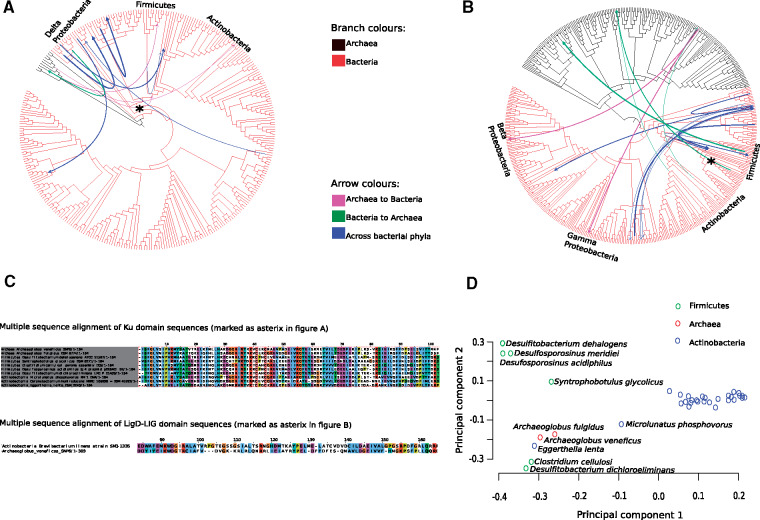
Extensive HGT among bacterial phyla and between bacteria and archaea. 16S rRNA-based species tree depicting the most frequent donor–recipient pairs involved in (*A*) Ku HGT events and (*B*) LigD-LIG HGT events. The bacterial species included in both the species tree coded for one Ku and one LigD only. The archaeal species were included 1) in (*A*) if they had at least a Ku and 2) in (*B*) if they had at least a LigD-LIG domain (see Materials and Methods for more details). The width of the arrow corresponds to the number of reconciliations supporting a given transfer event (see supplementary files 3 and 4, Supplementary Material online, for exact number of reconciliations for each donor–recipient pair). (*C*) Ku domain MSA (upper panel) of prokaryotes belonging to genus *Archaeoglobus* and phyla Firmicutes and Actinobacteria included in (*A*) (transfer event marked as asterisk). LigD-LIG pairwise alignment (lower panel) of an Archaea–Actinobacteria HGT transfer event in (*B*) (marked as asterisk). (*D*) Principal component analysis of Ku domain sequences evolved from ancestors included in transfer event in (*A*) (marked as asterisk).

An incongruence between a species and gene tree could result due to processes other than HGT, like duplications and losses. Therefore, to predict the most frequent transfer events, we used a reconciliation approach based on the DTL model. DTL employs a parsimonious framework where each evolutionary event is assigned a cost and the goal is to find a reconciliation (possible evolutionary history of gene tree inside a species tree) with minimum total cost. We observed a high rate of HGT between bacterial clades—Firmicutes, Actinobacteria, and Proteobacteria and Archaea, where each of these played the role of a donor and a recipient in Ku (fig. 6*A*) and LigD-LIG transfer events (fig. 6*B*). We observed that all proteobacterial species—with the exception of delta-proteobacteria, which was a donor of Ku to archaeal recipients—were recipients of both Ku and LigD from archaea or other distantly related bacteria. On one hand, we found evidence of Ku transfers from Archaea to Firmicutes and Actinobacteria and on the other hand, LigD-LIG transfers most likely occurred from Firmicutes and Actinobacteria to Archaea. Together, this raises the possibility of NHEJ transfers between bacteria and archaea.

An example of the former is depicted as a MSA of Ku domain sequences belonging to Archaea, Firmicutes, and Actinobacteria in figure 6*C* (upper panel). This transfer event corresponds to the asterisk marked in figure 6*A*—corresponding to that between the ancestor of the genus *Archaeoglobus* and that of Firmicutes and Actinobacteria. We further carried out a principal component analysis of these domain sequences that had evolved from the aforementioned ancestors (fig. 6*D*). Along the first principal component, all but two Actinobacteria—*Eggerthella lenta* and *Microlunatus phosphovorus*—form a distinct cluster from Archaea and Firmicutes. Along the second principal component, we see two distinct clusters. The cluster on the bottom left consists of anaerobic prokaryotes—Archaea (genus *Archaeoglobus*), Firmicutes (*Clostridium cellulosi* and *Desulfitobacterium dichloroeliminans*), and Actinobacteria (*Eggerthella lenta*), highlighting the possibility of HGT among these prokaryotes. Another instance of a LigD-LIG transfer is depicted in figure 6*C*, lower panel. This transfer event corresponds to the asterisk in figure 6*B*, involving the Actinobacteria—*Brevibacterium linens*, and Archaea—*Archaeoglobus veneficus* (coding for both Ku and LigD-LIG domains).

In addition to the evidence supporting HGT of NHEJ components between Archaea and Bacteria, we observed transfers among different bacterial clades as well (fig. 6*A* and *B*; blue arrows). We found Ku transfers between donor–recipient pairs: 1) Alphaproteobacterium (*Asticcacaulis excentricus*) and common ancestor of Acidobacteria (genus *Acidobacterium*, *Granulicella*, and *Terriglobus*) and 2) common ancestor of Delta-proteobacteria genus *Geobacter* and *Chlamydiae* (*Parachlamydia acanthamoeba*). For LigD-LIG transfers, we observed the following donor–recipient pairs—1) Proteobacteria (*Phenylbacterium zucineum*) and Acidobacteria (*Terriglobus roseus*) and 2) common ancestor of Actinobacteria (genus *Eggerthella)* and Firmicutes (*Desulfitobacterium dicholoroeliminans*). A full list of donor–recipient events can be found in supplementary files 3 and 4, Supplementary Material online, for Ku and LigD-LIG domains, respectively.

Kanhere and Vingron (2009) carried out HGT detection of bacterial core genes among prokaryotes. They found that a majority of these transfers occurred from bacteria to archaea and that these genes were mostly metabolic genes. Overall, our study is consistent with their observation, with additional evidence showing a possibility of NHEJ transfers from archaea to bacteria as well. We also show evidence of NHEJ transfers between closely and distantly related bacteria. Using the approach used in our study, it remains to be tested how HGT events have shaped noncore genes like other repair pathways throughout evolution in prokaryotes.

### NHEJ Occurrence Is Associated with GS, GR, and G–C Content

**Fig. 7:**
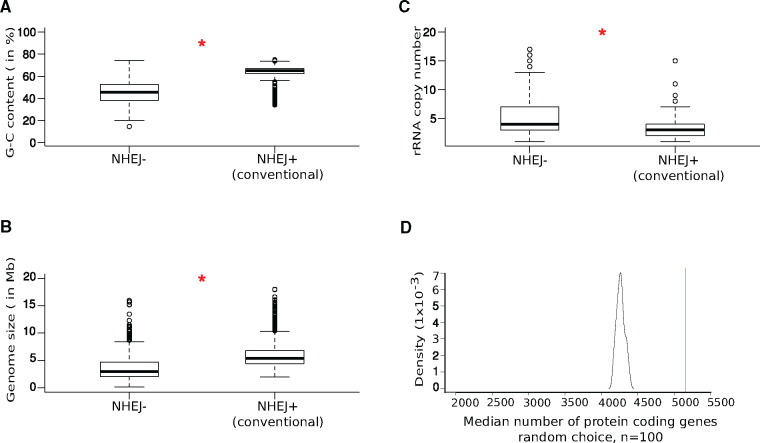
NHEJ presence and absence is associated with GS, GR, and G–C content. The red asterisk indicates statistical significance (Wilcoxon rank-sum test; *P* value < 0.01). (*A*) Boxplot comparing the distribution of G–C content between *NHEJ–* and *conventional NHEJ+* bacteria. (*B*) Boxplot comparing the distribution of GS between *NHEJ−* and *conventional NHEJ+* bacteria. (*C*) Boxplot comparing the distribution of rRNA copy number between *NHEJ−* and *conventional NHEJ+* bacteria. (*D*) A density distribution plot depicting the distribution of GS (median number of protein coding sequences) expected by a random distribution (black) where the probability of having NHEJ is linearly proportional to GS and the median GS of organisms harboring NHEJ (red).

Recently, Ku-encoding organisms were shown to have higher genomic G + C content (Weissman et al. 2019). Given its central role in DNA repair, we asked whether any other genome characteristics could also be associated with the presence or absence of NHEJ. First, we verified that the findings of Weissman et al. on the correlation between the presence of Ku and G + C content held true for *NHEJ+* states as defined in our study (fig. 7*A* and supplementary figs. S4, S5, and S7*C*, Supplementary Material online) (Weissman et al. 2019). Along with this, we tested two additional characteristics: GS and GR (as measured by the copy number of rRNA operons), both of which could determine the availability or the lack of a homologous template for high fidelity recombination-based repair. We restricted these analyses to conventional NHEJ-harboring bacteria as a proxy for repair proficiency and compared them with *NHEJ−* genomes. Data including nonconventional NHEJ are shown in supplementary figures S2 and S3, Supplementary Material online.

Bacteria with NHEJ were found to have larger genomes (median = 5.4 Mb) than those without NHEJ (median= 2.9 Mb; Wilcoxon rank-sum test, *P* < 10^−15^; fig. 7*B* and supplementary fig. S7*A*, Supplementary Material online) and significantly larger than that expected by a random distribution in which the probability of having NHEJ is linearly proportional to GS (Wilcoxon rank-sum test, *P* < 10^−15^, across 100 simulations; fig. 7*D*). This relationship was found to be true within the phylum Proteobacteria, Actinobacteria, Bacteroidetes, and Firmicutes as well (supplementary fig. S6, Supplementary Material online).

In addition, bacteria harboring NHEJ were found to have significantly fewer rRNA copies (median = 3), and by inference slower GRs, than bacteria without NHEJ (median = 4; Wilcoxon rank-sum test; *P* < 10^−15^; fig. 7*C*). Although the distribution of rRNA copy numbers for genomes without NHEJ was broad, those with conventional NHEJ fell within a narrow range, representing relatively slower growth (supplementary fig. S7*B*, Supplementary Material online). At the phyla level, this relationship was found to hold true for Proteobacteria and Actinobacteria, whereas there was no significant difference for Bacteroidetes and Firmicutes, respectively (supplementary fig. S8, Supplementary Material online).

In order to confirm the result in a phylogenetically controlled manner, Pagel’s *λ* and Blomberg’s *K* were used to first measure whether closely related bacteria tended to have similar GSs and GRs in the data set (see Materials and Methods). These measures suggest that phylogenetic coherence is significantly greater than random expectations for both the genome characteristics (supplementary table 9, Supplementary Material online). Therefore, the distributions of GS between bacteria with different NHEJ status were compared while accounting for the statistical nonindependence of closely related taxa (see Materials and Methods). We found a significant difference in log_10_(GSs) between bacteria with conventional NHEJ and without the repair (phyloANOVA; *P* = 6 × 10^−3^); the characters being mapped on the phylogenetic tree of 969 bacteria with five discrete groups: *NHEJ−*, *Ku only*, *LigD only*, *conventional NHEJ+*, and *nonconventional NHEJ+*. However, we did not observe a significant difference in log_10_(rRNA copy number) between the two groups of bacteria (phyloANOVA; *P* = 1; see Discussion).

We used ML as well as Bayesian approaches to test whether the observed associations of conventional NHEJ individually with large genomes and slow GRs are indicative of dependent or independent evolution of these traits on the phylogenetic tree (see Materials and Methods). Both suggested that the phylogenetic data fit models of evolution in which conventional NHEJ presence or absence and GS or GR are evolving in a correlated manner (table 1). This strengthens the association between the tested variables in a phylogenetically controlled way. Phylogenetic logistic regression of the conventional NHEJ occurrence with both the continuous independent variables showed, however, that GS is the stronger correlate (see Materials and Methods; table 2).

**Table 1:** Maximum Likelihood and Bayesian RJMCMC Results for Two Character Pairs Tested for Correlated Evolution: 1) NHEJ State and Genome Size and 2) NHEJ State and Growth Rate

Method	Correlation Pair	Log-Likelihood (Independent Model)	Marginal Log-Likelihood (Independent Model)	Log-Likelihood (Dependent Model)	Marginal Log-Likelihood (Dependent Model)	Likelihood Ratio Test (LRT) Statistics	Bayes Factor (>2 = Better Fit)
Maximum likelihood	NHEJ state and genome size	−1,059.97	—	−1,010.002	—	LR = 99.94 *P* < 0.001	—
Bayesian RJMCMC	NHEJ state and genome size	—	−1,110.14	—	−1,042.25	—	135.78
Maximum likelihood	NHEJ state and growth rate	−975.301	—	−950.01	—	LR = 25.29 *P* < 0.001	—
Bayesian RJMCMC	NHEJ state and growth rate	—	−1,051.529	—	−985.579		131.9

Note.—A chi-squared-based LRT with four degrees of freedom was used to test the better model—1) independent model where the character pair evolved independent of each other and 2) dependent model where the characters were allowed to evolve assuming a correlated evolution—based on maximum likelihood. Bayes factor was used to test the better model, based on Bayesian RJMCMC analysis.

**Table 2:** Phylogenetic Logistic Regression for Three Models, Based on a Phylogenetic Tree of 1,403 Species of Bacteria Harboring Either *NHEJ−* or *Conventional NHEJ+* State

Model	AIC Value	Pen.LogLik	Alpha	Coefficient Estimate (CE)	*P* Value
NHEJ ∼ GS	809.5	−383.3.	13.88	6.7 × 10^−7^	10^−15^
NHEJ ∼ GR	916.7	−450.1	23.85	−0.133	7 × 10^−4^
NHEJ = GS + GR	841.2	−395.1	23.51	CE_GS_ = 8.1 × 10^−7^ CE_GR_ = −0.4	10^−15^ 3.8 × 10^−12^

Note.—Alpha represents the phylogenetic signal of the dependent variable, that is, NHEJ state in our case. The higher the alpha, the lesser the phylogenetic signal. AIC or the Akaike Information Criterion is used to select the best model for the NHEJ state, out of the three tested against two independent variables—GS and GR. The two independent variables were tested against multicollinearity, with variance inflation factor or VIF = 0.93715 (VIF < 10 is preferred), making the analysis reliable.

As a case study where the association of both GS and GR with NHEJ evolution was prominent, we found a gain of conventional NHEJ in the ancestor of two genera belonging to Corynebacteriales—*Mycobacterium* and *Corynebacterium*—where the former retained and the latter had a secondary loss of the machinery. Phylogenetic ancestral reconstruction analysis revealed an increase in GS in the ancestor of Corynebacteriales, followed by an NHEJ gain. Although *Mycobacterium* retained NHEJ, *Corynebacterium* lost the machinery along with a decrease in GS. Using a similar analysis, GR mapped to this subclade revealed an increase in rRNA copy number in *Corynebacterium* (supplementary fig. S9, Supplementary Material online; see Materials and Methods).

## Discussion

Taking advantage of the availability of a large number of genomes, we confirm the nonubiquitous nature of NHEJ across the bacterial domain (Aravind and Koonin 2001; Matthews and Simmons 2014) with 22% bacteria coding for it. At the taxa level, although some phyla retain this sporadicity, others are devoid of NHEJ repair machinery, consistent with previous studies on distribution of bacterial NHEJ proteins (Matthews and Simmons 2014). In line with previous reports on the multiplicity of NHEJ in certain bacteria (Bétermier et al. 2014; Hoff et al. 2016, 2018; McGovern et al. 2016; Dupuy et al. 2017, 2019), we further discovered a sporadic presence of multiple copies of NHEJ proteins extending to different subclades of Proteobacteria, Actinobacteria, Firmicutes, Bacteroidetes, etc. (supplementary table 1, Supplementary Material online). A trace of the evolutionary history suggested that NHEJ was most likely absent in the eubacterial ancestor. Furthermore, 96% archaeal genomes coded for LIG but no Ku. Therefore, by inference, NHEJ was absent in the Most Recent Common Ancestor of all prokaryotes as well. It was instead gained independently multiple times in different bacterial lineages. However, these primary gains were not sufficient to stabilize the repair states in subclades, because a large number of secondary losses and gains were observed across the phylogeny (elaborated further below).

Our analysis suggests that there are two common methods to arrive at an *NHEJ+* state: 1) the most common way to gain NHEJ was by the acquisition of LigD followed by Ku and 2) a direct transition from an *NHEJ−* to *NHEJ+* state. We found that the LigD only state is more prevalent and is a prominent intermediate in the evolution of the *NHEJ+* state in Proteobacteria and Bacteroidetes (pp_Proteobacterial-subclade_ = 0.875 and pp_Bacteroidetes_ = 0.7; supplementary fig. S10*C* and *D*, Supplementary Material online). However, 90% of *LigD only* genomes did not encode POL and PE domains, raising the possibility that *LigD only* to *NHEJ+* transitions could actually be *NHEJ−* to *NHEJ+* transitions.

Direct NHEJ gain can be explained by their acquisition via HGT. In this direction, we observed 60 *NHEJ+* organisms coding this repair on their plasmids. However, our analysis based on the detection of base compositional differences revealed no horizontally acquired chromosomal NHEJ. These numbers may be an underestimate because one is unlikely to pick HGT events if 1) the regions getting horizontally transferred have the same compositional biases in the donor and the recipient cells and/or 2) such HGT events occur early in bacterial evolution. Two instances of the latter case that we observe in our analysis are direct primary gains of NHEJ at the ancestral nodes of Firmicutes and Actinobacteria, with high posterior probability support (pp_Firmicutes_ = 0.987 and pp_Actinobacteria_ = 0.967; supplementary fig. S10*A* and *B*, Supplementary Material online). Aravind and Koonin (2001) proposed a possibility of HGT between bacteria and archaea. In this direction, using a gene tree-species tree reconciliation approach, we observed evidence for HGT between bacteria and archaea. Furthermore, this method allowed us to find evidence of HGT between distantly related bacteria as well (supplementary tables 3 and 4, Supplementary Material online). It should be noted that for the lack of methods to accurately date bacterial species trees and because transfers from unsampled species or extinct lineages could violate time constraints, we used an undated phylogeny for this analysis. Therefore, for each most frequent donor predicted, we noted the most frequent recipient as the potential donor–recipient pair. A similar search for NHEJ components in ∼3,000 actinobacteriophages, yielded no hits, although, Pitcher et al. (2006) have shown the employment of *Mycobacterium smegmatis* LigD by the Ku expressing mycobacteriophages Omega and Corndog for a successful infection. Thus, although our results suggest no transfer events between bacteria and bacteriophages, whether this is an artifact of sequence amelioration processes (Lawrence and Ochman 1997) remains to be tested.

A third route to an *NHEJ+* state could have been via a *Ku only* state. However, we observed that the time spent in a *Ku only* state is the least and NHEJ gains via this route are rare. Approximately 90% *Ku only* states are present in Firmicutes alone, specifically belonging to two genera, *Bacillus* and *Fictibacillus*. This suggests that *Ku only* state is largely avoided across most bacteria. This could be because Ku alone is nonfunctional in repair or in some cases could even block the access of break ends for recombination-based repair (Sinha et al. 2009; Gupta et al. 2011; Zhang et al. 2012). In 138 organisms, where Ku is retained in the absence of LigD, its function could be mediated by cross-talk with other ligases such as LigA. Consistent with this, it has been previously shown that if damage produces 3′ overhangs specifically, LigA could repair the lesion even in the absence of LigB/C/D (Aniukwu et al. 2008). In vitro studies have also shown that *Mycobacterium smegmatis* Ku can stimulate T4 DNA ligase (Kushwaha and Grove 2013). We observed the same trend in Archaea; Ku was always present with a LIG domain in ten archaeal species, whereas 230 archaea coded for LIG domain and no Ku. These observations are consistent with experimental studies reporting a fully functional complement of NHEJ being present only in a single archaea, and with the likelihood of microhomology-mediated end joining (via ligase alone) being more prevalent in these organisms (Bartlett et al. 2013; White and Allers 2018).

Furthermore, we observed that a primary gain does not stabilize the NHEJ trait in the subsequent lineages; with the most common type of loss being *NHEJ+* to *NHEJ−*. We discuss this, with examples, at two levels—1) across genera belonging to closely related phyla and 2) within the same genera. As discussed earlier, there was a direct primary gain of NHEJ at the common ancestor of the phyla Firmicutes and Tenericutes (fig. 4). First, we found that the monophyletic class Mollicutes (belonging to Tenericutes), which includes genera like the obligate plant parasites *Phytoplasma* and human parasites *Mycoplasma*, have had a one-step secondary loss of NHEJ during their evolution. This is consistent with the well supported hypothesis that these Mollicutes have evolved from Gram-positive bacteria including Firmicutes by reductive genome evolution (Woese et al. 1980; Wolf et al. 2004; Oshima et al. 2013; Ipoutcha et al. 2019). This suggests that NHEJ is either dispensable or costly to maintain a parasitic lifestyle in this class of bacteria. Second, although many species of the para- and poly-phyletic genus *Clostridium* (belonging to Firmicutes) including *Clostridium cellulosi* and *Clostridium stercorarium* retained NHEJ, others like *Clostridium clariflavum* and *Clostridium propionicum* had direct secondary losses. Furthermore, species like *Clostridium phytofermantans* and *Clostridium saccharolyticum* had direct secondary gains. A full list of NHEJ primary/secondary gains and losses at different taxa level—direct and sequential—can be found in the supplementary table 8, Supplementary Material online.

It is likely that several factors have contributed to the current distribution of NHEJ in bacterial systems. For example: 1) studies in both prokaryotes and eukaryotes have suggested a cross-talk between NHEJ and other repair mechanisms like recombination-based repair (Hoff et al. 2016, 2018; Biehs et al. 2017; Bertrand et al. 2019), base excision repair (Bertrand et al. 2019), and mismatch repair (Shen et al. 2018). Shen et al. (2018) have shown that a bacteriophage infection is prevented by generating DSBs produced by MutL, a conventional mismatch repair endonuclease, which is subsequently repaired by NHEJ via a large deletion. However, this might not be a conserved pathway of repair as our analysis suggests an absence of NHEJ in a majority of actinomycetes belonging to the genus *Corynebacterium* including *Corynebacterium glutamicum* and 96% of archaea. 2) NHEJ could co-occur with other pathways that may or may not be directly involved in DSB repair. For example, NHEJ has been found to be active during sporulation in *Bacillus subtilis* (Wang et al. 2006; Bertrand et al. 2019), however, nonspore-forming bacteria also encode for NHEJ (Aravind and Koonin 2001; McGovern et al. 2016). Additional cross-talk at the level of regulation has also been reported, such as in *Mycobacterium*, where the deacetylase Sir2 has been implicated in regulating NHEJ (Li et al. 2011, p. 2). It is equally plausible that signatures of displacement of certain domains from genomes encoding NHEJ could inform us on how acquisition of NHEJ could have shaped genome architecture. In line with this, we did note 20 additional domains coded in Ku and LigD proteins (supplementary table 10, Supplementary Material online) across different phyla. These would be useful to experimentally test in the future to understand whether they show functional interaction with NHEJ repair.

To understand the selection pressures that might play a role in shaping the evolutionary pattern of NHEJ, we focused our efforts on studying the genome characteristics associated with this DNA repair. We reasoned that under the event of a DSB, the unavailability of a template would prevent the highly accurate homologous recombination repair to act. Thus, factors that affect rates of genome duplication or probability of multiple DSBs occurring could be central to determining the need for NHEJ-based repair. Therefore, one might expect a higher selection pressure of maintaining NHEJ in bacteria with slower GRs and larger GSs. In line with our hypothesis, we found that the organisms possessing conventional NHEJ tend to have significantly larger GS and slower GR as compared with those that are devoid of it. A phylogenetically controlled test suggested that this association held true for GS and not GR. We note that phyloANOVA used here for hypothesis testing assumes normality. However, our data for GS and rRNA copy number are not normally distributed, even after converting them into a logarithmic scale (Shapiro–Wilk normality test; *P*_genome size_ = 1.7 × 10^−10^, *P*_rRNA copy number_ < 10^−15^). Therefore, the result should be interpreted keeping this caveat in mind. Furthermore, ML and Bayesian inferences showed that there is a correlated evolution of NHEJ with GS and GR along the phylogenetic tree. Considering the two variables together, we found that GS is the better correlate of the *NHEJ+* state. Therefore, we conclude that the *NHEJ+* state is strongly associated with GS and to a much smaller extent with GR throughout its evolution.

In sum, our study highlights the evolutionary trajectory of NHEJ and central characteristics that may have determined its sporadic distribution. DSB repair, including NHEJ, has been implicated in shaping bacterial genomes through mutagenesis (Paris et al. 2015), HGT (Popa et al. 2011), and their effect on genomic G–C content (Weissman et al. 2019). Given this relationship between repair and genome evolution, it is important to ask how one factor may have influenced the other during bacterial evolution. It is also possible that similar forces have played a role in the evolution of other repair pathways and the genomes encoding them.

## Supplementary Material

Supplementary data are available at *Genome Biology and Evolution* online.

## Supplementary Material

evaa223_Supplementary_Data
