# Supplementary material for: Evolutionary and Comparative Analysis of Bacterial Nonhomologous End Joining Repair
Source: Genome Biol Evol. 2020 Oct 20;12(12):2450–66. doi: 10.1093/gbe/evaa223 (PMC7719229; doi:10.1093/gbe/evaa223)
Supplement: evaa223_Supplementary_Data [file evaa223_supplementary_data.zip › Supplementary_figures_w_legends.pdf]

Figure S1

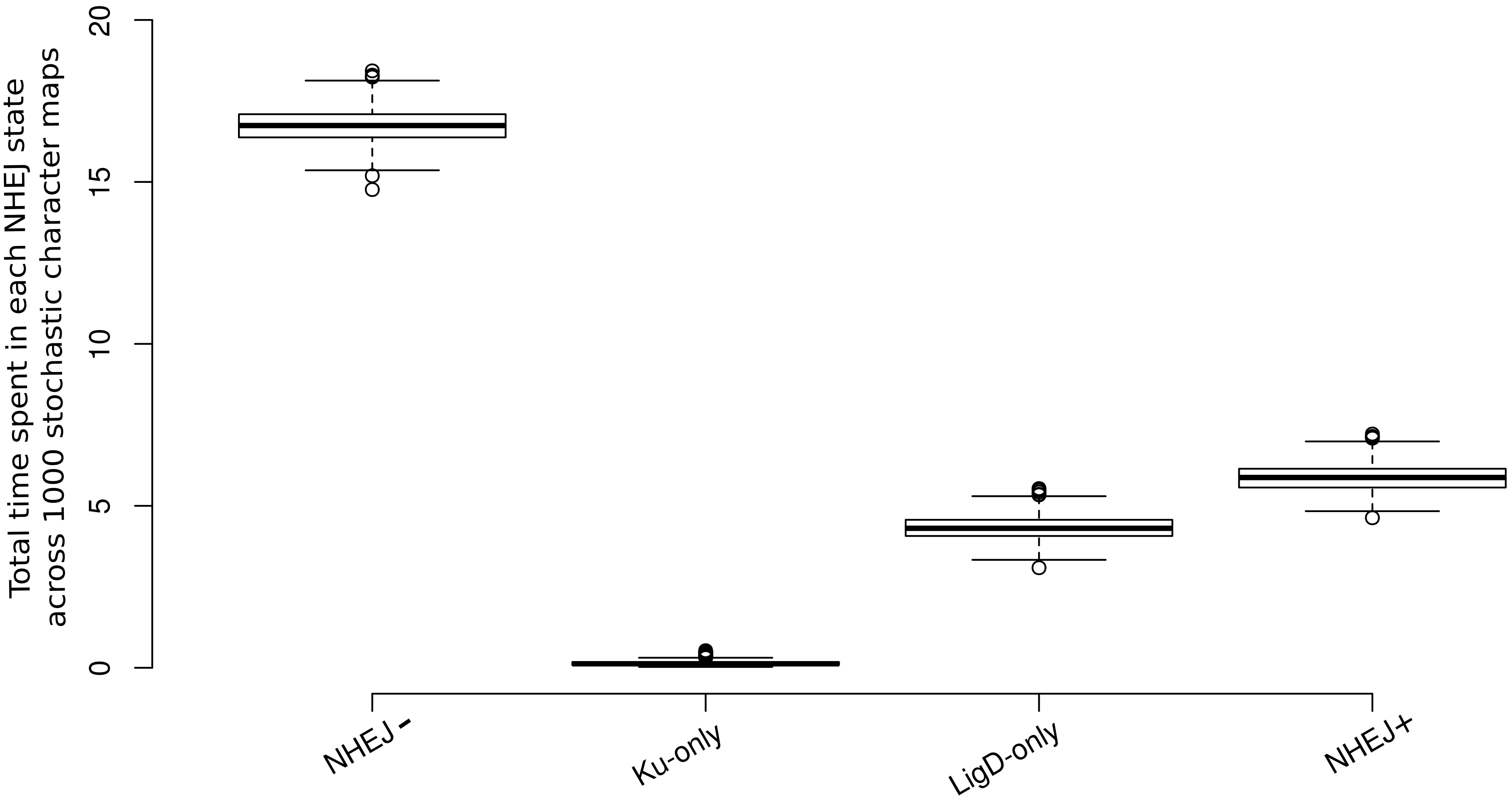
Total time spent in each NHEJ state
across 1000 stochastic character maps
0
5
10
15
20
NHEJ -
Ku-only
LigD-only
NHEJ+

Figure S2

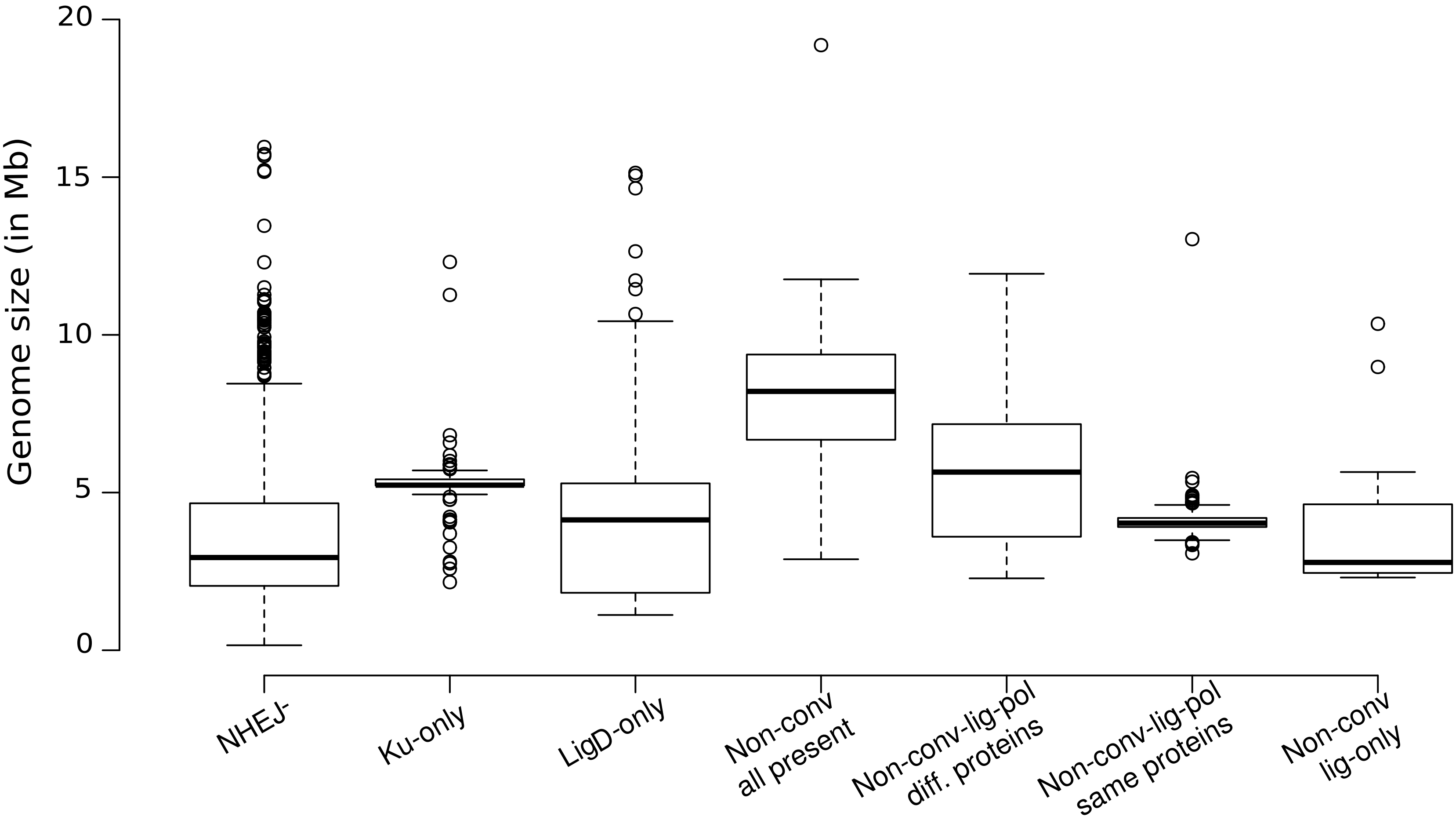

Genome size (in Mb)
0
5
10
15
20
NHEJ-
Ku-only
LigD-only
Non-conv
all present
Non-conv-lig-pol
diff. proteins
Non-conv-lig-pol
same proteins
Non-conv
lig-only

Figure S3

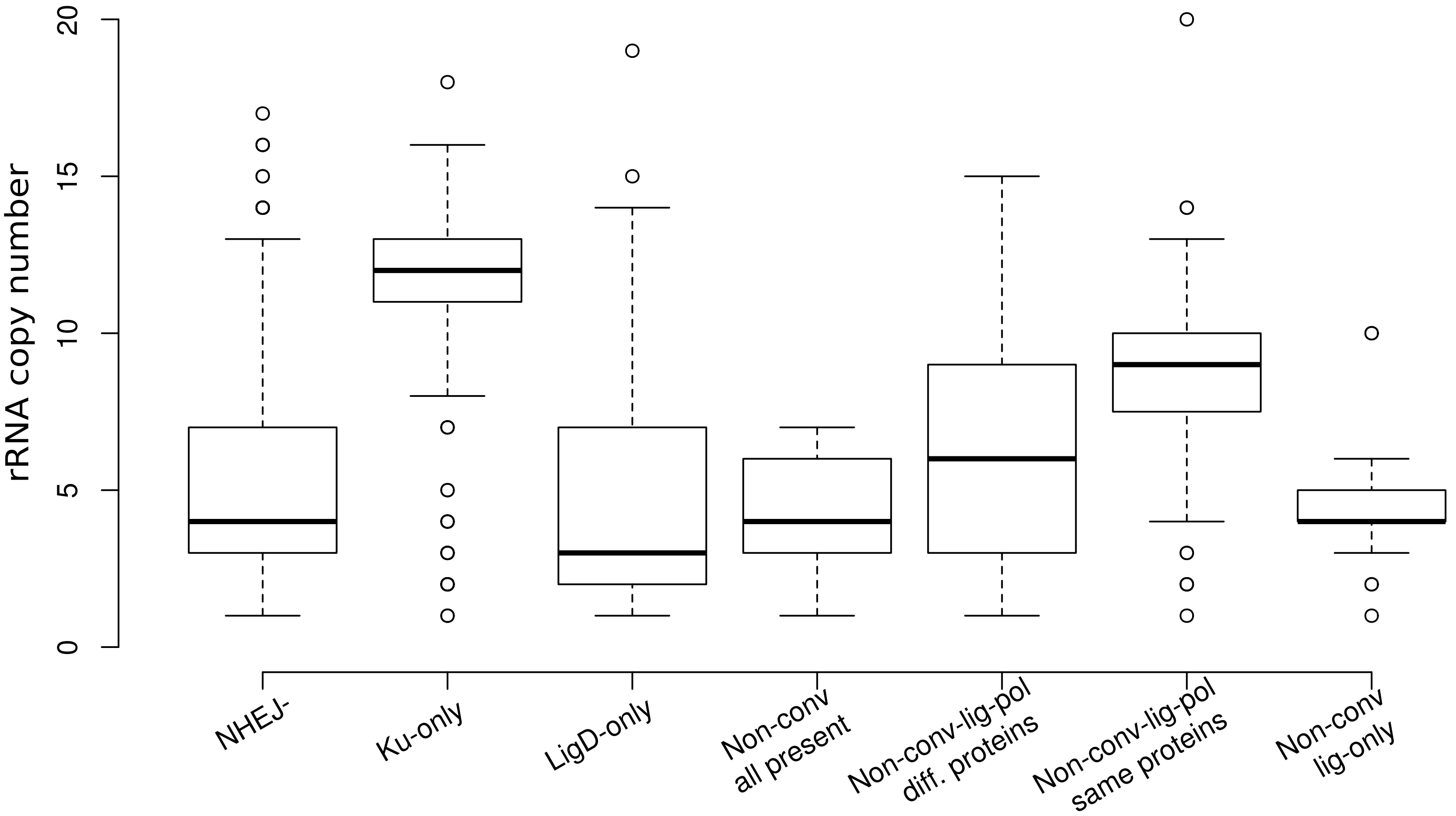
rRNA copy number
0
5
10
15
20
NHEJ-
Ku-only
LigD-only
Non-conv
all present
Non-conv-lig-pol
diff. proteins
Non-conv-lig-pol
same proteins
Non-conv
lig-only

Figure S4

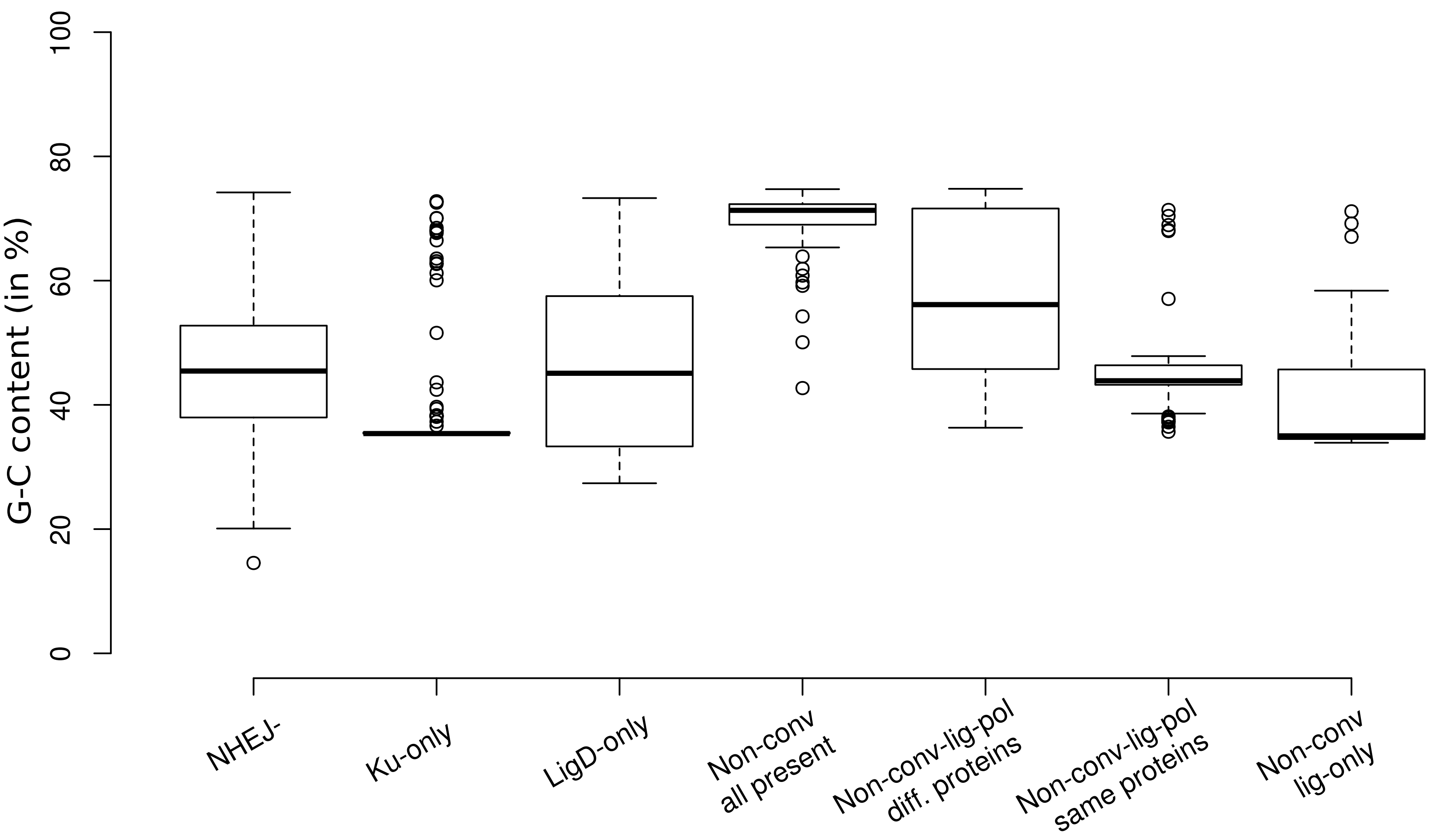
G-C content (in %)
0
20
40
60
80
100
NHEJ-
Ku-only
LigD-only
Non-conv
all present
Non-conv-lig-pol
diff. proteins
Non-conv-lig-pol
same proteins
Non-conv
lig-only

Figure S5

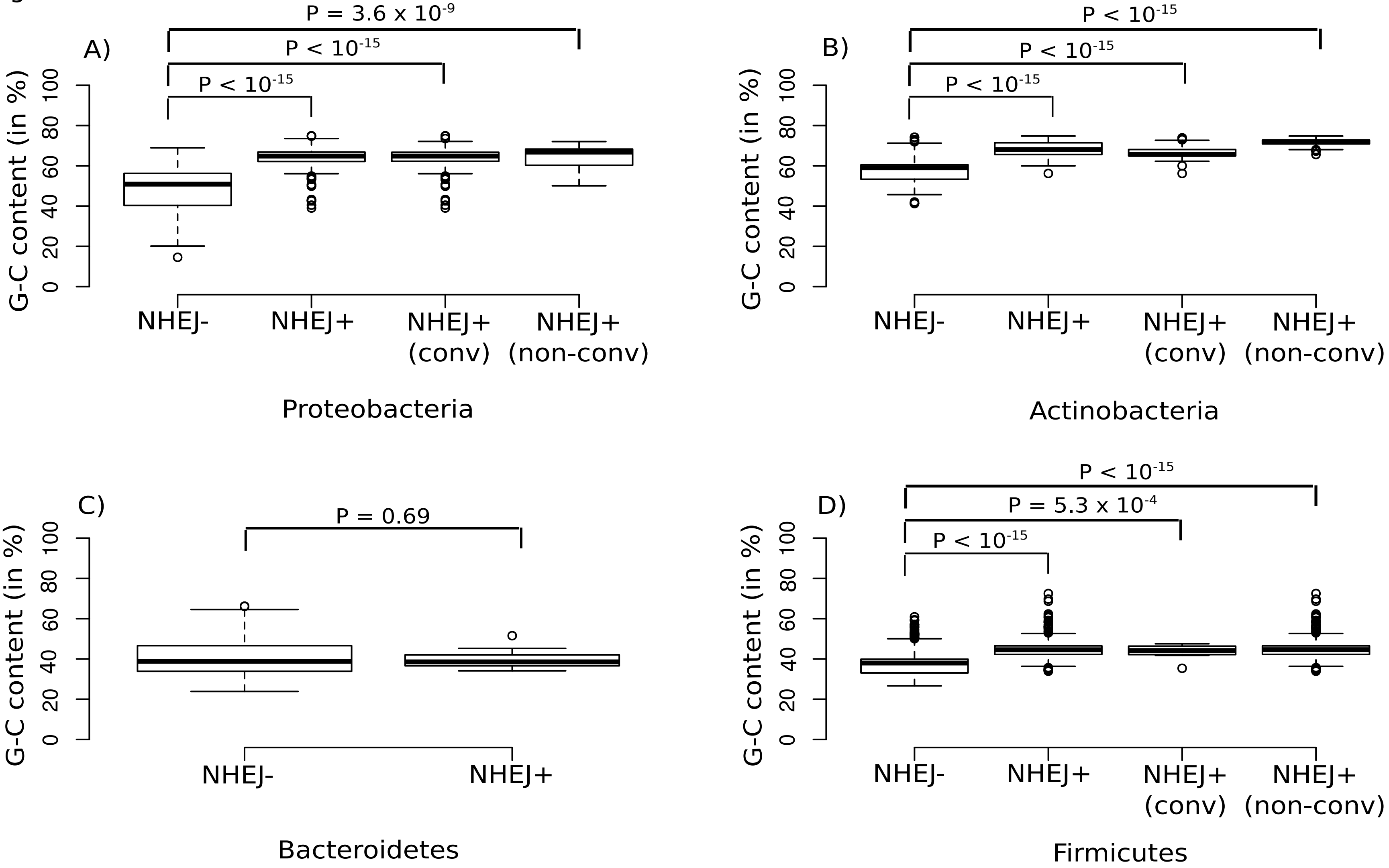

A)
P = 3.6 x 10-9
P < 10-15
P < 10-15
G-C content (in %)
0
20
40
60
80
100
NHEJ-
NHEJ+
NHEJ+ (conv)
NHEJ+ (non-conv)
Proteobacteria
B)
P < 10-15
P < 10-15
P < 10-15
G-C content (in %)
0
20
40
60
80
100
NHEJ-
NHEJ+
NHEJ+ (conv)
NHEJ+ (non-conv)
Actinobacteria
C)
P = 0.69
G-C content (in %)
0
20
40
60
80
100
NHEJ-
NHEJ+
Bacteroidetes
D)
P < 10-15
P = 5.3 x 10-4
P < 10-15
G-C content (in %)
0
20
40
60
80
100
NHEJ-
NHEJ+
NHEJ+ (conv)
NHEJ+ (non-conv)
Firmicutes

Figure S6

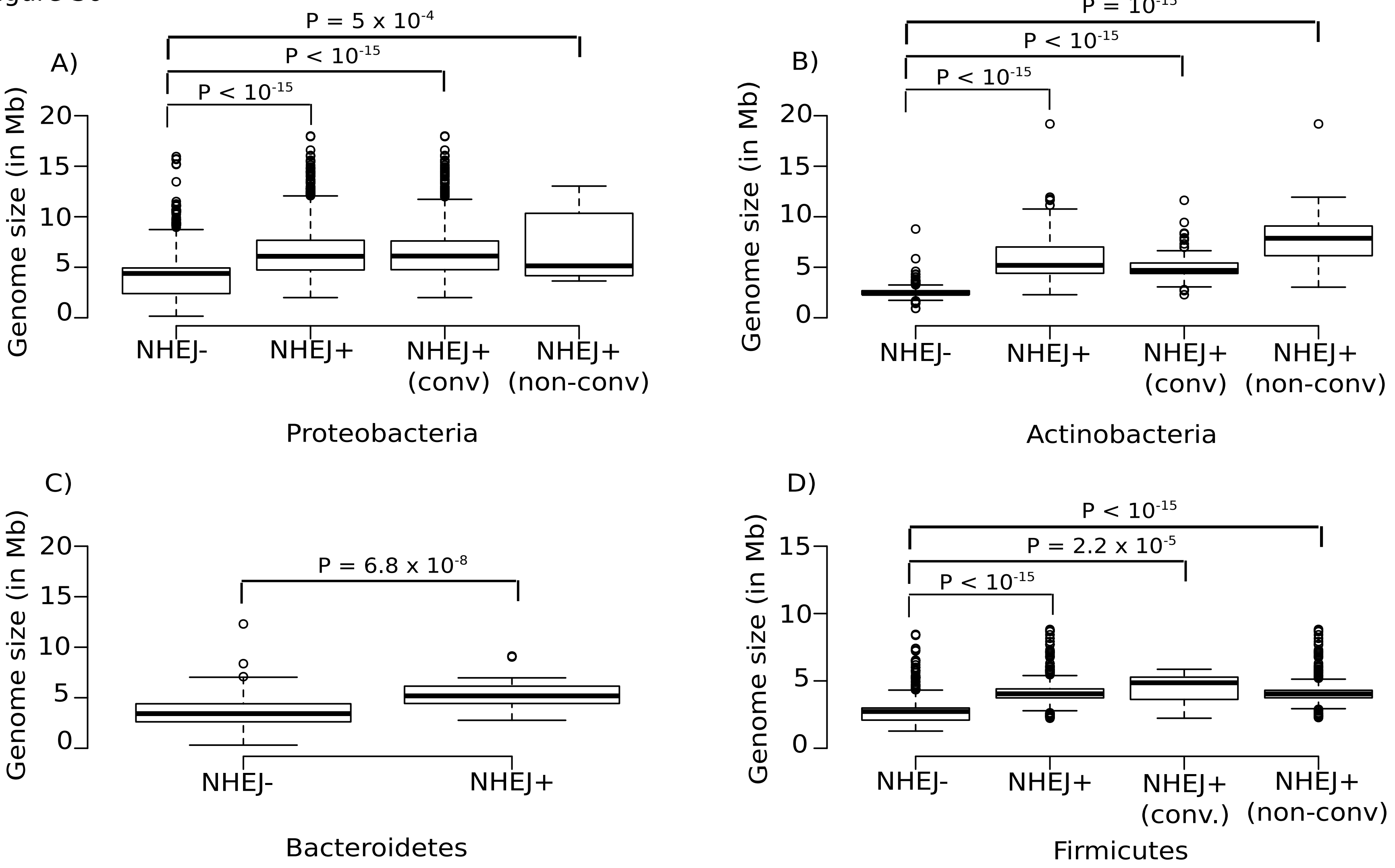

A)
P = 5 x 10-4
P < 10-15
P < 10-15
Genome size (in Mb)
20
15
10
5
0
NHEJ-
NHEJ+
NHEJ+ (conv)
NHEJ+ (non-conv)
Proteobacteria
B)
P = 10-15
P < 10-15
P < 10-15
Genome size (in Mb)
NHEJ-
NHEJ+
NHEJ+ (conv)
NHEJ+ (non-conv)
Actinobacteria
C)
P = 6.8 x 10-8
Genome size (in Mb)
NHEJ-
NHEJ+
Bacteroidetes
D)
P < 10-15
P = 2.2 x 10-5
P < 10-15
Genome size (in Mb)
NHEJ-
NHEJ+
NHEJ+ (conv.)
NHEJ+ (non-conv)
Firmicutes

Figure S7

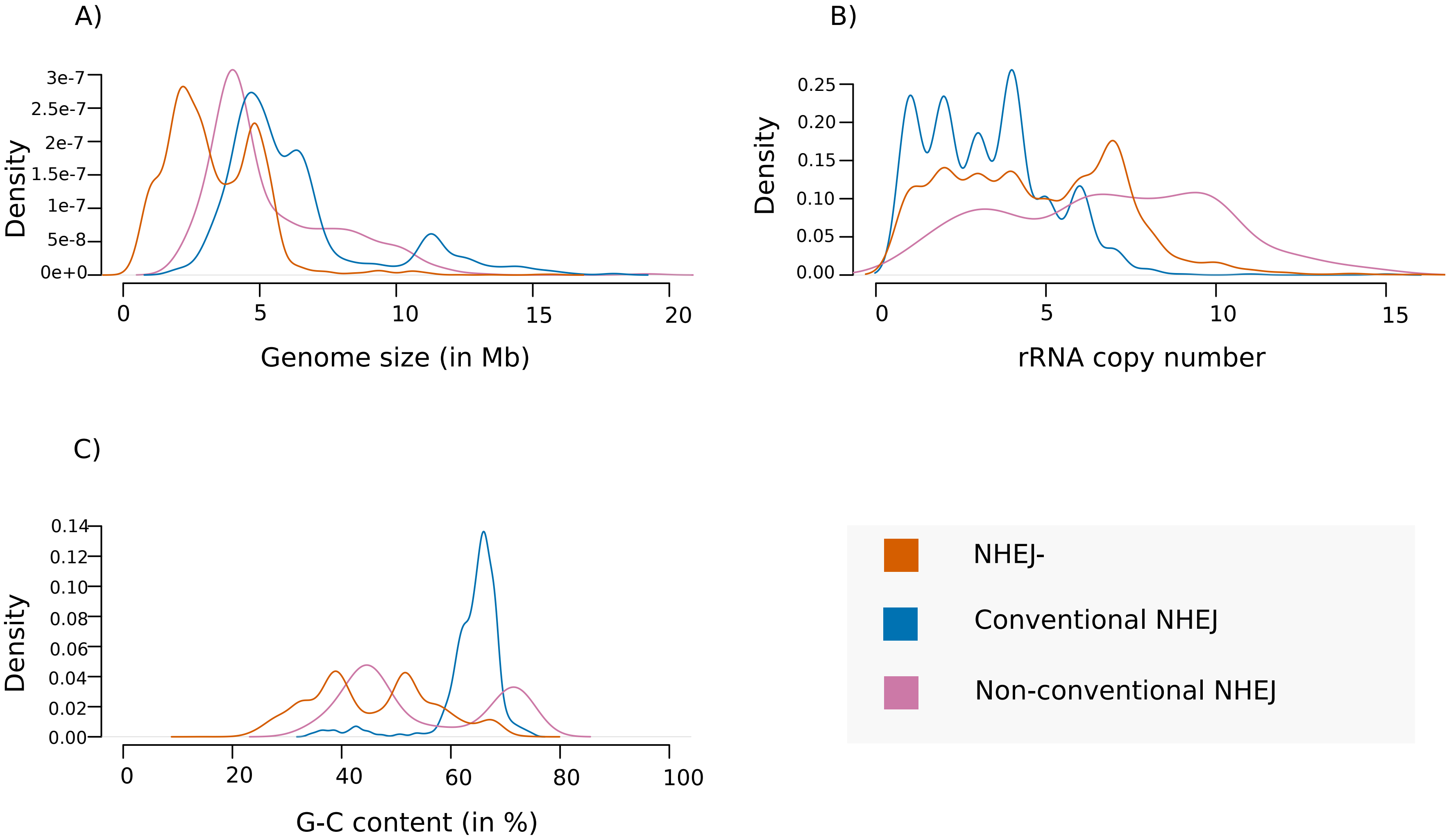
A)
Density
3e-7
2.5e-7
2e-7
1.5e-7
1e-7
5e-8
0e+0
0
5
10
15
20
Genome size (in Mb)
B)
Density
0.25
0.20
0.15
0.10
0.05
0.00
0
5
10
15
rRNA copy number
C)
Density
0.14
0.12
0.10
0.08
0.06
0.04
0.02
0.00
0
20
40
60
80
100
G-C content (in %)
NHEJ-
Conventional NHEJ
Non-conventional NHEJ

Figure S8

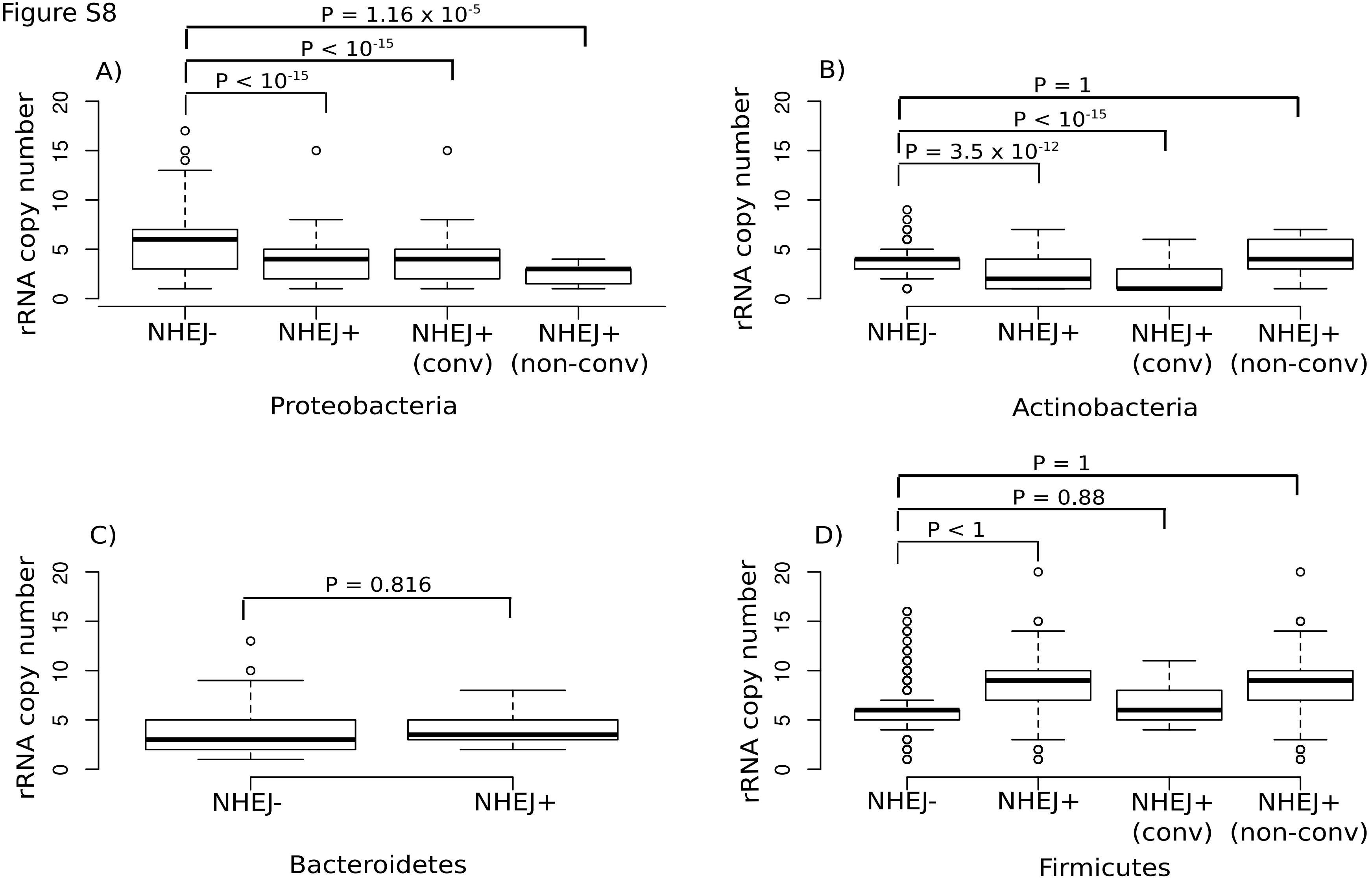

A)
P = 1.16 x 10-5
P < 10-15
P < 10-15
rRNA copy number
0
5
10
15
20
NHEJ-
NHEJ+
NHEJ+ (conv)
NHEJ+ (non-conv)
Proteobacteria
B)
P = 1
P < 10-15
P = 3.5 x 10-12
rRNA copy number
0
5
10
15
20
NHEJ-
NHEJ+
NHEJ+ (conv)
NHEJ+ (non-conv)
Actinobacteria
C)
P = 0.816
rRNA copy number
0
5
10
15
20
NHEJ-
NHEJ+
Bacteroidetes
D)
P = 1
P = 0.88
P < 1
rRNA copy number
0
5
10
15
20
NHEJ-
NHEJ+
NHEJ+ (conv)
NHEJ+ (non-conv)
Firmicutes

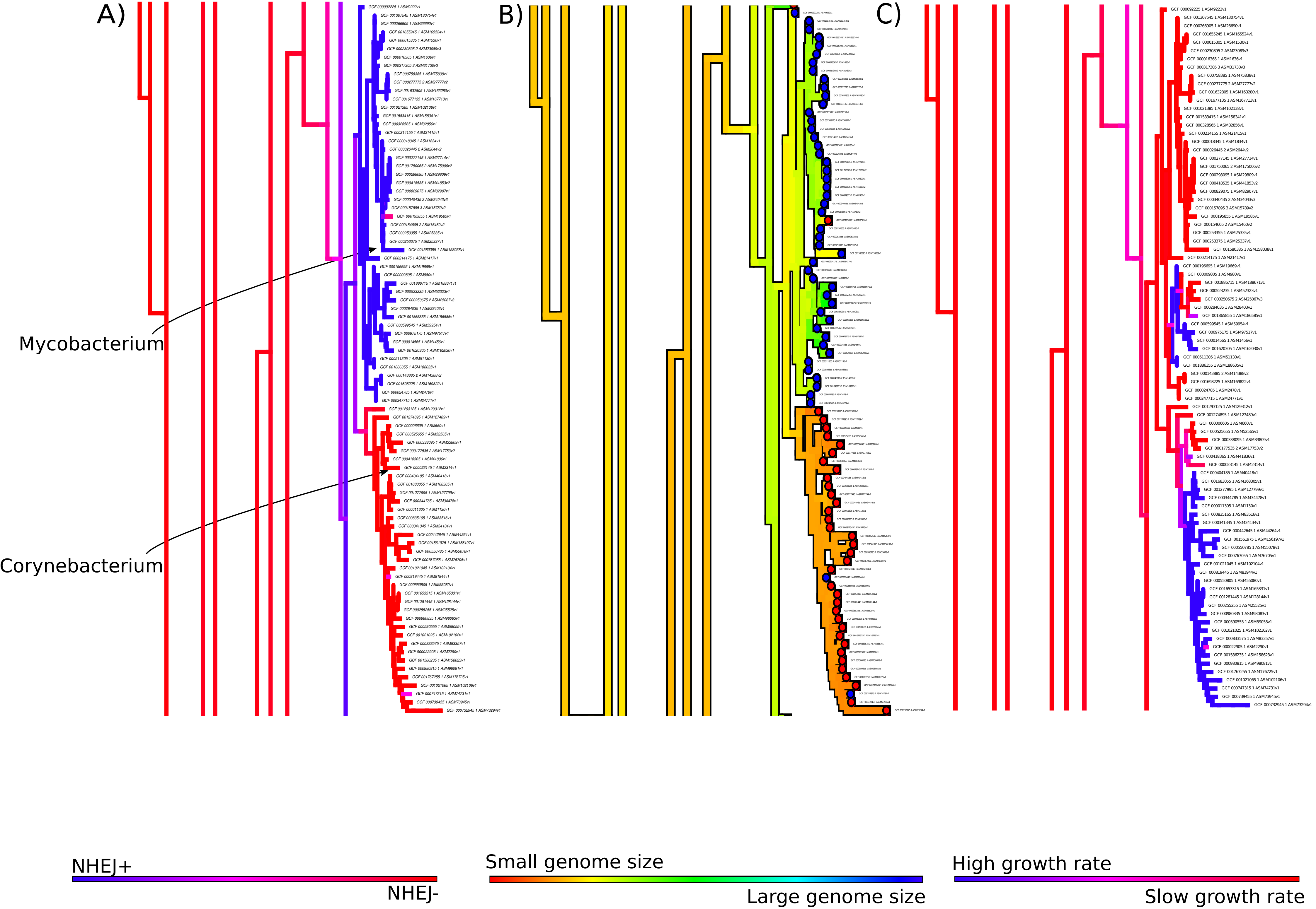
A)
B)
C)
Mycobacterium
Corynebacterium
NHEJ+
NHEJ-
Small genome size
Large genome size
High growth rate
Slow growth rate

Figure S10

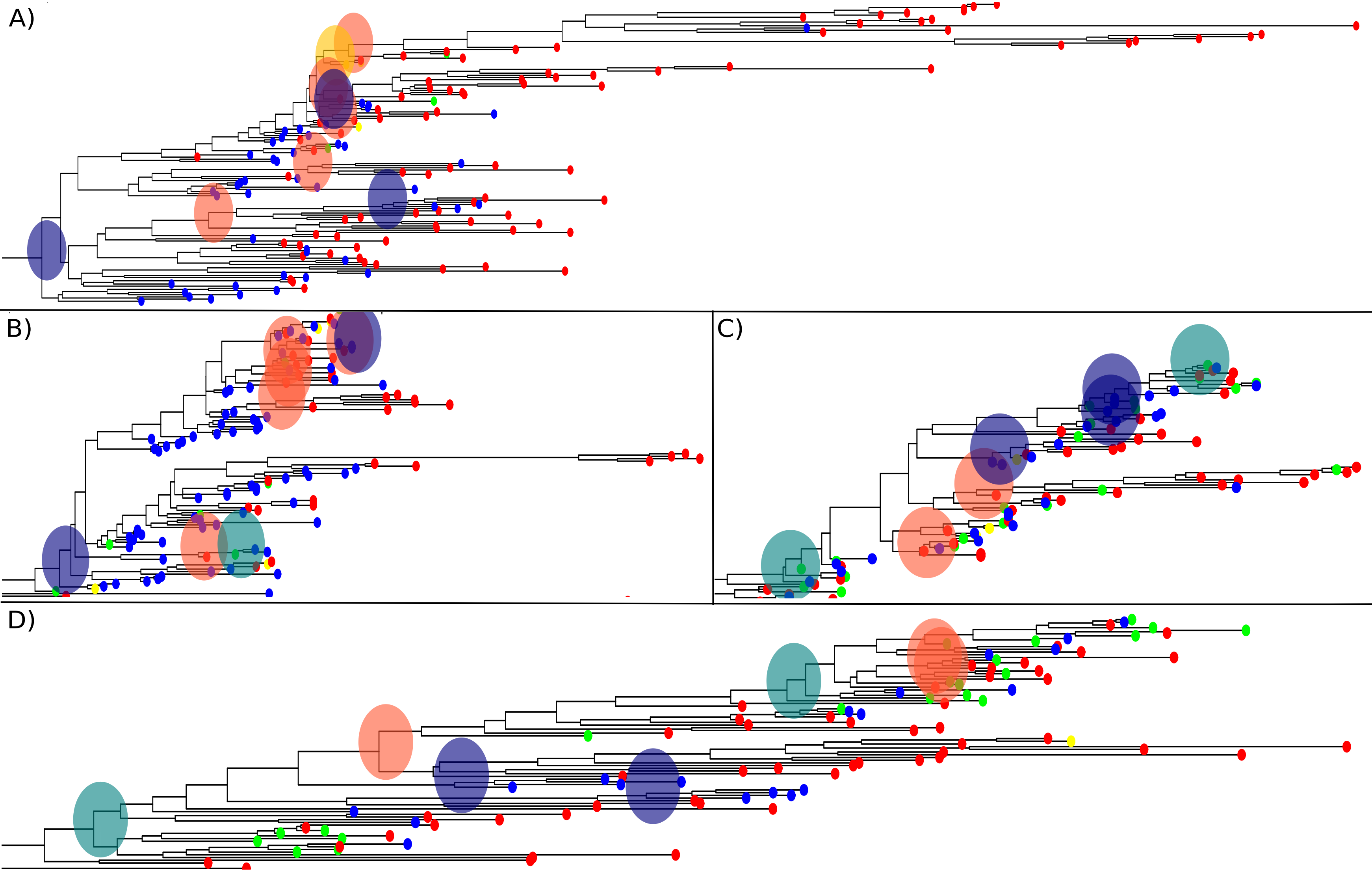

Supplementary figure legends

Figure S1: Boxplots representing the distribution of total time spent in a given NHEJ state over a phylogenetic tree across 1000 stochastic character maps (Y-axis). X-axis represents the four NHEJ states: *NHEJ-*, *Ku only*, *LigD only* and *NHEJ+*.

Figure S2: Boxplots representing distribution of genome sizes in bacteria with different combinations of NHEJ machinery present: 1) *NHEJ-*, 2) *Ku only*, 3) *LigD only*, 4) *Non-conventional NHEJ+* (all domains present excluding the conventional LigD), 5) *Non-conventional NHEJ+* (LIG and POL domain present in different proteins and no PE domain), 6) *Non-conventional NHEJ+* (LIG and POL domain present in the same protein and no PE domain) and 7) *Non-conventional NHEJ+* (LIG domain present and no POL domain)

Figure S3: Boxplots representing distribution of rRNA copy numbers in bacteria with different combinations of NHEJ machinery present: 1) *NHEJ-*, 2) *Ku only*, 3) *LigD only*, 4) *Non-conventional NHEJ+* (all domains present excluding the conventional LigD), 5) *Non-conventional NHEJ+* (LIG and POL domain present in different proteins and no PE domain), 6) *Non-conventional NHEJ+* (LIG and POL domain present in the same protein and no PE domain) and 7) *Non-conventional NHEJ+* (LIG domain present and no POL domain)

Figure S4: Boxplots representing distribution of G-C content in bacteria with different combinations of NHEJ machinery present: 1) *NHEJ-*, 2) *Ku only*, 3) *LigD only*, 4) *Non-conventional NHEJ+* (all domains present excluding the conventional LigD), 5) *Non-conventional NHEJ+* (LIG and POL domain present in different proteins and no PE domain), 6) *Non-conventional NHEJ+* (LIG and POL domain present in the same protein and no PE domain) and 7) *Non-conventional NHEJ+* (LIG domain present and no POL domain)

Figure S5: Boxplots representing distribution of G-C content in bacteria with different NHEJ states - *NHEJ-* , *conventional NHEJ+* and *non-conventional NHEJ+* across four phyla. G-C content is significantly higher in organisms with NHEJ as compared to those that lack it in three phyla – A) Proteobacteria, B) Actinobacteria and D) Firmicutes. There is no significance difference in G-C content in C) Bacteroidetes.

Figure S6: Boxplots representing distribution of genome size in bacteria with different NHEJ states - *NHEJ-* , *conventional NHEJ+* and *non-conventional NHEJ+* across four phyla. Genome size is significantly higher in organisms with NHEJ as compared to those that lack it in all four phyla – A) Proteobacteria, B) Actinobacteria, C) Bacteroidetes and D) Firmicutes.

Figure S7: Density plots representing the distribution of A) Genome size, B) rRNA copy number and C) G-C content across bacteria with different NHEJ states- *NHEJ-* (orange), *Conventional NHEJ+* (blue) and *Non-conventional NHEJ+* (pink).

Figure S8: Boxplots representing distribution of rRNA copy number in bacteria with different NHEJ states - *NHEJ-* , *conventional NHEJ+* and *non-conventional NHEJ+* across four phyla. rRNA copy number is significantly lower in organisms with NHEJ as compared to those that lack it in two phyla – A) Proteobacteria, B) Actinobacteria. There is no significant difference in rRNA copy number distributions in C) Bacteroidetes. In D) Firmicutes, organisms harboring NHEJ (conventional and non-

conventional) tend to have significantly higher growth rate than organisms without NHEJ. However, when NHEJ + organisms are segregated into conventional and non-conventional, we find no significant difference in rRNA copy numbers between *NHEJ–* and *conventional NHEJ+* Firmicutes.

Figure S9: A) Density map of phylogenetic ancestral reconstruction of conventional NHEJ presence (blue) and absence (red), a binary trait, represented as posterior probability across 1000 stochastic maps in two Corynebacteriales sub-clades: Mycobacterium and Corynebacterium. B) Density map of phylogenetic ancestral reconstruction of genome size, a continuous trait, using the Brownian motion model. The warmer colours represent smaller genome size and colder colours represent larger genome sizes. The tip colours represent conventional NHEJ presence (blue) and absence (red) in the contemporaneous species. C) Density map of phylogenetic ancestral reconstruction of the growth rate, high (blue) and slow (red), a binary trait, represented as posterior probability across 1000 stochastic maps.

Figure S10: A) Direct primary NHEJ gain at the ancestral node of Firmicutes, Fusobacteria and Tenericutes (shallowest phylogenetic depth). B) Direct primary NHEJ gain at the ancestral node of Actinobacteria. C) Sequential primary gain of LigD followed by Ku to yield NHEJ in sub-clade of Proteobacteria. D) Sequential primary gain of LigD in the ancestral node of Bacteroidetes, followed by the complete gain of NHEJ by the acquisition of Ku in different sub-clades. Tip and Node colours-Red: *NHEJ-* , Blue: *NHEJ+* , Green: *LigD only* , Yellow: *Ku only*.
