# Supplementary material for: Evolutionary and Comparative Analysis of Bacterial Nonhomologous End Joining Repair
Source: Genome Biol Evol. 2020 Oct 20;12(12):2450–66. doi: 10.1093/gbe/evaa223 (PMC7719229; doi:10.1093/gbe/evaa223)
Supplement: evaa223_Supplementary_Data [file evaa223_supplementary_data.zip › Supplementary_files_7_8_9.pdf]

Supplementary table 7
Contingency tables and Fisher's Exact test results, run on 969 organisms:

!Conventional NHEJ = Non-conventional NHEJ + No-NHEJ
!Non-conventional NHEJ = Conventional NHEJ + No-NHEJ

	Proteobacteria	! Proteobacteria
Conventional NHEJ	98	58
! Conventional NHEJ	368	446

```
Fisher's Exact Test for Count Data

data:  prot.conventional
p-value = 3.803e-05
alternative hypothesis: true odds ratio is greater than 1
95 percent confidence interval:
 1.501099      Inf
sample estimates:
odds ratio
  2.046295
```

	Proteobacteria	! Proteobacteria
Non-Conventional NHEJ	15	91
! Non-Conventional NHEJ	451	413

```
Fisher's Exact Test for Count Data

data:  prot.nonconventional
p-value = 1
alternative hypothesis: true odds ratio is greater than 1
95 percent confidence interval:
 0.08864445        Inf
sample estimates:
odds ratio
  0.151218
```

	Actinobacteria	! Actinobacteria
Conventional NHEJ	28	128
! Conventional NHEJ	716	98

```
Fisher's Exact Test for Count Data

data:  actino.conventional
p-value = 1
alternative hypothesis: true odds ratio is greater than 1
95 percent confidence interval:
 0.01976974        Inf
sample estimates:
odds ratio
0.03013512
```

	Actinobacteria	! Actinobacteria

Non-Conventional NHEJ	44	62
! Non-Conventional NHEJ	82	782

```
Fisher's Exact Test for Count Data

data:  actino.nonconventional
p-value = 2.123e-15
alternative hypothesis: true odds ratio is greater than 1
95 percent confidence interval:
 4.511277      Inf
sample estimates:
odds ratio
  6.745681
```

	Bacteroidetes	! Bacteroidetes
Conventional NHEJ	19	137
! Conventional NHEJ	74	740

```
Fisher's Exact Test for Count Data

data:  bacteroidetes.conventional
p-value = 0.1468
alternative hypothesis: true odds ratio is greater than 1
95 percent confidence interval:
 0.8406495       Inf
sample estimates:
odds ratio
   1.38635
```

	Bacteroidetes	! Bacteroidetes
Non-Conventional NHEJ	0	106
! Non-Conventional NHEJ	93	771

```
Fisher's Exact Test for Count Data

data:  bacteroidetes.nonconventional
p-value = 1
alternative hypothesis: true odds ratio is greater than 1
95 percent confidence interval:
   0 Inf
sample estimates:
odds ratio
         0
```

	Firmicutes	! Firmicutes
Conventional NHEJ	5	151
! Conventional NHEJ	122	692

```
Fisher's Exact Test for Count Data

data:  firmicutes.conventional
p-value = 1
```

```
alternative hypothesis: true odds ratio is greater than 1
95 percent confidence interval:
 0.07174517        Inf
sample estimates:
odds ratio
 0.1880178
```

	Firmicutes	! Firmicutes
Non-Conventional NHEJ	42	64
! Non-Conventional NHEJ	85	779

```
Fisher's Exact Test for Count Data

data:  firmicutes.nonconventional
p-value = 1.23e-13
alternative hypothesis: true odds ratio is greater than 1
95 percent confidence interval:
 4.00504     Inf
sample estimates:
odds ratio
  5.997159
```

	Acidobacteria	! Acidobacteria
Conventional NHEJ	3	153
! Conventional NHEJ	3	811

```
Fisher's Exact Test for Count Data

data:  acidobacteria.conventional
p-value = 0.05622
alternative hypothesis: true odds ratio is greater than 1
95 percent confidence interval:
 0.947979      Inf
sample estimates:
odds ratio
  5.286889
```

	Acidobacteria	! Acidobacteria
Non-Conventional NHEJ	2	104
! Non-Conventional NHEJ	4	860

```
Fisher's Exact Test for Count Data

data:  acidobacteria.nonconventional
p-value = 0.1325
alternative hypothesis: true odds ratio is greater than 1
95 percent confidence interval:
 0.5472948       Inf
sample estimates:
odds ratio
  4.125139
```

	Chlamydiae	! Chlamydiae
Conventional NHEJ	1	155
! Conventional NHEJ	5	809

```
Fisher's Exact Test for Count Data

data:  chlamydiae.conventional
p-value = 0.6518
alternative hypothesis: true odds ratio is greater than 1
95 percent confidence interval:
 0.04453014        Inf
sample estimates:
odds ratio
  1.043831
```

	Chlamydiae	! Chlamydiae
Non-Conventional NHEJ	1	105
! Non-Conventional NHEJ	5	859

```
Fisher's Exact Test for Count Data

data:  chlamydiae.nonconventional
p-value = 0.5015
alternative hypothesis: true odds ratio is greater than 1
95 percent confidence interval:
 0.06959043        Inf
sample estimates:
odds ratio
  1.635181
```

	Verrucomicrobia	! Verrucomicrobia
Conventional NHEJ	0	156
! Conventional NHEJ	6	808

```
Fisher's Exact Test for Count Data

data:  verrucomicrobia.conventional
p-value = 1
alternative hypothesis: true odds ratio is greater than 1
95 percent confidence interval:
   0 Inf
sample estimates:
odds ratio
         0
```

	Verrucomicrobia	! Verrucomicrobia
Non-Conventional NHEJ	1	105
! Non-Conventional NHEJ	5	859

```
Fisher's Exact Test for Count Data

data:  verrucomicrobia.nonconventional
p-value = 0.5015
alternative hypothesis: true odds ratio is greater than 1
95 percent confidence interval:
 0.06959043        Inf
sample estimates:
odds ratio
  1.635181
```

<u>List of phyla with all *No-NHEJ* state organisms only:</u>

1) Tenericutes
2) Thermotogae
3) Synergistetes
4) Fusobacteria
5) Chlorobi
6) Deferribacteres
7) Elusimicrobia
8) Ignavibacteriae
9) Fibrobacteres
10) Coprothermobacterota
11) Chrysiogenetes
12) Calditrichaeota
13) Kirtimatiellaeota
14) Candidatus Cloacimonetes

<u>List of phyla with *incomplete NHEJ* or *No-NHEJ* state organisms only:</u>

1) Caldiserica
2) Dictyoglomi
3) Gemmatimonadetes
4) Thermobaculum
5) Deinococcus-Thermus
6) Planctomycetes
7) Aquificae
8) Chloroflexi
9) Cyanobacteria
10) Spirochaetes

Supplementary table 8

	Sub-phyla name	Type of gain	Type of loss	Organism names
	Flavobacteriia	Sequential secondary gain (NHEJ- to LigD to NHEJ+)	-	Maribacter sp. 1_2014MBL_MicDiv; Cellulophaga baltica NN016038; Flavobacterium johnsoniae UW101; Aequorivita sublithincola DSM 14238; Zunongwangia profunda SM-A87; Gramella sp. LPB0144
		Secondary direct gain (NHEJ- to NHEJ+)	-	Chryseobacterium sp. IHB B17019; Flavobacteriaceae bacterium 3519-10
	Common ancestor of Flavobacteriia and Bacteroidia	-	Major Secondary loss of LigD to NHEJ-	-
	Sphingobacteria	Primary sequential gain (LigD to NHEJ+)	-	Mucilaginibacter sp. PAMC 26640 plasmid unnamed; Pedobacter saltans DSM 12145; Sphingobacterium sp. ML3W; Solitalea canadensis DSM 3403
	Chitinophagia	Primary NHEJ sequential gain (LigD to NHEJ+)	-	Niabella ginsenosidivorans strain BS26; Niastella koreensis GR20−10; Flavisolibacter sp. LCS9; Chitinophaga pinensis DSM 2588
	Cytophagia	Primary NHEJ sequential gain (LigD to NHEJ+)	-	Cytophaga hutchinsonii ATCC 33406; Dyadobacter fermentans DSM 18053; Cyclobacterium amurskyense strain KCTC 12363
	Common ancestor of Flavobacteriia, Bacteroidia, Sphingobacteria, Chitinophagia and a subclade of Cytophagia	Major Primary LigD gain	-	-

	Thermoleophilia	Minor Primary direct gain (NHEJ- to NHEJ+)	-	Conexibacter woesei DSM 14684
	Coriobacteriia	Minor Primary direct gain (NHEJ- to NHEJ+)	-	Eggerthella lenta DSM 2243
	Acidimicrobiia	Minor Primary direct gain (NHEJ- to NHEJ+)	-	Ilumatobacter coccineus YM16−304
	Common ancestor of Frankiales, Geodermatophilales, Streptosporangiales, Streptomycetales, Catenulisporales, Glycomycetales, Pseudonocardiales, Propionibacteriales, Corynebacteriales, Micromonosporales, Bifidobacteriales, Micrococcales, Kineosporiales, Nakamurellales, Actinomycetales	Major Primary direct gain	-	
	Frankiales	Primary direct gain		Frankia sp. EuI1c; Frankia sp. EAN1pec
	Geodermatophilales	Primary direct gain		Blastococcus saxobsidens DD2; Geodermatophilus obscurus DSM 43160; Modestobacter marinus str. BC501
	Actinobacteria incertae sedis	Primary direct gain		Thermobispora bispora DSM 43833
	Streptosporangiales	Primary direct gain		Thermomonospora curvata DSM 43183; Streptosporangium roseum DSM 43021
	Streptosporangiales	-	Direct secondary loss	Thermobifida fusca; Nocardiopsis alba ATCC BAA−2165
	Catenulisporales	Primary direct gain		Catenulispora acidiphila DSM 44928
	Streptomycetales	-	Direct secondary loss	Kitasatospora setae KM−6054 DNA
	Streptomycetales	Primary direct gain	-	Streptomyces lydicus strain 103;

				Streptomyces sampsonii strain KJ40
	Pseudonocardiales	Primary direct gain	-	Actinoalloteichus hymeniacidonis strain HPA177(T) (=DSM 45092(T)); Pseudonocardia dioxanivorans CB1190; Kibdelosporangium phytohabitans strain KLBMP1111; Saccharothrix espanaensis DSM; Alloactinosynnema sp. L−07; Actinosynnema mirum DSM 43827; Lentzea guizhouensis strain DHS C013; Saccharopolyspora erythraea NRRL2338; Saccharomonospora viridis DSM 43017; Amycolatopsis japonica strain MG417−CF17;
	Glycomycetales	Primary direct gain	-	Stackebrandtia nassauensis DSM 44728;
	Corynebacteriales	Primary direct gain	-	Rhodococcus fascians D188; Nocardia seriolae strain EM150506; Corynebacterium humireducens NBRC 106098 = DSM 45392; Mycobacterium tuberculosis 49−02; Mycobacterium goodii strain 7B; Amycolicicoccus subflavus DQS3−9A1; Gordonia sp. QH−11; Gordonia sp. KTR9; Tsukamurella paurometabola DSM 20162;
	Corynebacteriales	-	Direct secondary loss	Brevibacterium flavum ZL−1; Corynebacteriales bacterium 1698; Segniliparus rotundus DSM 44985; Corynebacterium pseudotuberculosis strain 36;

				Mycobacterium leprae TN
	Propionibacteriales	Direct primary gain	-	Microlunatus phosphovorus NM−1 DNA;<br>Nocardioides dokdonensis FR1436;<br>Pimelobacter simplex strain VKM Ac−2033D;<br>Aeromicrobium erythreum strain AR18;<br>Kribbella flavida DSM 17836
	Propionibacteriales	-	Direct secondary loss	Cutibacterium avidum strain DPC 6544;<br>Propionibacterium acnes C1
	Bifidobacteriales	-	Direct secondary loss	Scardovia inopinata JCM 12537 DNA;<br>Parascardovia denticolens DSM 10105 = JCM 12538 DNA;<br>Gardnerella vaginalis ATCC 14018 = JCM 11026 DNA;<br>Bifidobacterium breve 12L
	Micromonosporales	Direct primary gain	-	Salinispora arenicola CNS−205;<br>Salinispora tropica CNB−440;<br>Micromonospora sp. L5;<br>Verrucosispora maris AB−18−032;<br>Actinoplanes friuliensis DSM 7358
	Nakamurellales	Direct primary gain	-	Nakamurella multipartita DSM 44233
	Kineosporiales	Direct primary gain	-	Kineococcus radiotolerans SRS30216 plasmid pKRAD02
	Micrococcales	Direct primary gain	-	Xylanimonas cellulosilytica DSM 15894 ;<br>Isoptericola dokdonensis DS−3;<br>Beutenbergia cavernae DSM 12333;<br>Serinicoccus sp. JLT9;<br>Kytococcus sedentarius DSM 20547;<br>Dermacoccus

				nishinomiyaensis strain M25; Luteipulveratus mongoliensis strain MN07−A0370; Arsenicicoccus sp. oral taxon 190; Intrasporangium calvum DSM 43043; Janibacter terrae strain | F|001
	Micrococcales	-	Direct secondary loss	Jonesia denitrificans DSM 20603; Tropheryma whipplei TW08/27
	Actinomycetales	-	Direct secondary loss	Trueperella pyogenes TP8; Arcanobacterium haemolyticum DSM 20595; Actinobaculum schaalii strain CCUG 27420; Actinomyces sp. Marseille−P2985 strain Marseille−P2985T ; Mobiluncus curtisii ATCC 43063;
	Micrococcales	Direct primary gain	-	Brevibacterium linens strain SMQ−1335; Sanguibacter keddieii DSM 10542; Cellvibrio gilvus ATCC 13127; Cellulomonas fimi ATCC 484; Brachybacterium faecium DSM 4810; Sinomonas atrocyanea strain KCTC 3377; Arthrobacter aurescens TC1 plasmid TC2; Rathayibacter tritici strain NCPPB 1953; Agromyces aureus strain AR33; Microbacterium sp. 1.5R; Microbacterium sp. T11; Cryobacterium arcticum strain PAMC 27867; Curtobacterium sp. BH−2−1−1; Frondihabitans sp. SR6 plasmid 3; Clavibacter

				michiganensis subsp. insidiosus strain R1−1; Leifsonia xyli strain SE134
	Micrococcales	-	Direct secondary loss	Devriesea agamarum genome assembly S2_rcS3_S1_rcS3; Rothia mucilaginosa D| −18 DNA; Kocuria rhizophila DC2201; Dermabacter vaginalis strain AD1−86; Neomicrococcus aestuarii strain B18; Arthrobacter sp. A3; Renibacterium salmoninarum ATCC 33209; Micrococcus luteus NCTC 2665; Microbacterium sp. No. 7; Rathayibacter toxicus strain WAC3373; Microterricola viridarii strain ERGS5:02
	Fimbriimonadia	Direct primary gain	-	Fimbriimonas ginsengisoli Gsoil 348
	Clostridia	Direct primary gain	-	Thermaerobacter marianensis DSM 12885; Candidatus Desulforudis audaxviator MP104C;
	Thermodesulfobacteriales	Direct primary gain	-	Thermodesulfatator indicus DSM 15286
	Clostridia	Direct primary gain	-	Thermosediminibacter oceani DSM 16646; Thermacetogenium phaeum DSM 12270; Carboxydothermus hydrogenoformans Z−2901; Syntrophothermus lipocalidus DSM 12680; Syntrophomonas wolfei subsp. wolfei str. Goettingen G311; Moorella thermoacetica strain DSM 103132; Natranaerobius thermophilus

				JW/NM−WN−LF; Thermoanaerobacter sp. 513; Halothermothrix orenii H 168; Limnochorda pilosa DNA; Symbiobacterium thermophilum IAM 14863 DNA; Clostridium cellulosi genome assembly DG5; Clostridium stercorarium subsp. stercorarium DSM 8532; Clostridium stercorarium subsp. leptospartum DSM 9219; Thermoanaerobacterium thermosaccharolyticum M0795; Desulfotomaculum reducens MI−1; Thermincola sp. JR; Desulfitobacterium dehalogenans ATCC 51507; Desulfitobacterium metallireducens DSM 15288; Syntrophobotulus glycolicus DSM 8271; Heliobacterium modesticaldum Ice1 strain Ice1; Desulfosporosinus meridiei DSM 13257; Dehalobacter restrictus DSM 9455; Pelotomaculum thermopropionicum SI DNA
	Clostridia	-	Direct Secondary loss	Tepidanaerobacter acetatoxydans Re1; Caldicelulosiruptor becscii DSM 6725; Caldicellulosiruptor saccharolyticus DSM 8903; Ethanoligenens harbinense |UAN−3; Ruminococcus bicirculans; Halobacteroides halobius DSM 5150; Acetohalobium

				arabaticum DSM 5501; Halanaerobium praevalens DSM 2228; Flavonifractor plautii strain |L31; Intestinimonas butyriciproducens strain AF211; Oscillibacter valericigenes Sjm18−20; Mahella australiensis 50−1 BON; Ruminiclostridium thermocellum DSM 2360; Clostridium clariflavum DSM 19732; Clostridiales genomosp. BVAB3 str. UPII9−5; Eubacterium limosum strain SA11; Acetobacterium woodii DSM 1030; Desulfotomaculum acetoxidans DSM 771
	Common ancestor of Tissierellia and a sub-clade of Clostridia	-	Direct Secondary loss	
	Clostridia	-	Direct Secondary loss	Clostridium propionicum DSM 1682; Peptoclostridium difficile strain 08ACD0030; Filifactor alocis ATCC 35896; Geosporobacter ferrireducens strain IRF9; Alkaliphilus metalliredigens Q|MF; Lachnoclostridium sp. | L32; Butyrivibrio proteoclasticus B316; Roseburia hominis A2−183
	Clostridia	Direct Secondary gain	-	Blautia sp. |L58; Clostridium saccharolyticum WM1; Clostridium phytofermentans ISDg

	Tissierellia	-	Direct Secondary loss	Levyella sp. Marseille−P3170 strain Marseille−P3170T ; Murdochiella sp. Marseille−P2341 strain Marseille−P2341T ; Parvimonas micra strain KCOM 1535; Finegoldia magna ATCC 29328; Peptoniphilus sp. ING2−D1G; Anaerococcus prevotii DSM 20548
	Negativicutes	-	Direct Secondary loss	Acidaminococcus fermentans DSM 20731; Selenomonas sputigena ATCC 35185; Dialister pneumosintes strain F0677; Megasphaera elsdenii 14−14; Veillonella parvula DSM 2008
		Direct Secondary gain	-	Pelosinus fermentans JBW45
	Bacilli	Direct Primary gain	-	Kyrpidia tusciae DSM 2912; Alicyclobacillus acidocaldarius subsp. acidocaldarius Tc−4−1; Thermobacillus composti KWC4; Bacillus cellulosilyticus DSM 2522; Paenibacillus sp. LPB0068; Paenibacillus polymyxa SC2; Halobacillus halophilus DSM 2266; Terribacillus aidingensis strain MP602; Lentibacillus amyloliquefaciens strain LAM0015; Oceanobacillus iheyensis HTE831; Virgibacillus sp. 6R; Bacillus pseudofirmus OF4; Lysinibacillus sphaericus strain 2362; Solibacillus silvestris strain DSM 12223; Rummeliibacillus stabekisii strain PP9;

		-	Direct Secondary loss	Geobacillus stearothermophilus 10; Paenibacillus sp. BD3526; Salimicrobium jeotgali strain MJ3; Amphibacillus xylanus NBRC 15112; Planococcus rifietoensis strain M8; Anoxybacillus sp. B7M1; Exiguobacterium antarcticum B7; Sporosarcina psychrophila strain DSM 6497; Aneurinibacillus sp. H2; Kurthia sp. 11Kri321; Jeotgalibacillus sp. D5; Leuconostoc carnosum JB16; Oenococcus oeni PSU−1; Weissella koreensis KACC 15510; Pediococcus damnosus strain TMW 2.1534; Aerococcus christensenii strain CCUG28831; Lactobacillus salivarius strain JCM 1046; Marinilactibacillus sp. 15R; Carnobacterium sp. WN1359; Streptococcus constellatus subsp. pharyngis C818; Lactococcus garvieae Lg2 DNA; Enterococcus faecium strain 64/3; Melissococcus plutonius S1; Vagococcus teuberi strain DSM21459T; Tetragenococcus halophilus NBRC 12172 DNA; Listeria monocytogenes strain ATCC 19117; Macrococcus caseolyticus JCSC5402; Salinicoccus halodurans

				strain H3B36; Bacillus cereus subsp. cytotoxis NVH 391−98; Gemella sp. oral taxon 928; Staphylococcus aureus strain CA15
		Direct Secondary gain	-	Brevibacillus laterosporus LMG 15441
	Erysipelotrichia	Direct Secondary gain	-	Erysipelotrichaceae bacterium I46
		-	Direct Secondary loss	Turicibacter sp. H121; Erysipelothrix rhusiopathiae str. Fujisawa DNA; Faecalibaculum rodentium strain Alo17;
	Fusobacteriales	-	Direct Secondary loss	Ilyobacter polytropus DSM 2926; Fusobacterium nucleatum subsp. animalis strain KCOM 1279v; Sebaldella termitidis ATCC 33386; Leptotrichia sp. oral taxon 847; Streptobacillus moniliformis DSM 12112; Sneathia sp. Sn35;
	Mollicutes	-	Direct Secondary loss	Strawberry lethal yellows phytoplasma (CPA) str. NZSb11; Aster yellows witches'−broom phytoplasma A|WB; Maize bushy stunt phytoplasma strain M3; Onion yellows phytoplasma O|−M DNA; Acholeplasma palmae; Acholeplasma oculi; Acholeplasma oculi strain 19L; Mollicutes bacterium HR1; Mycoplasma mycoides subsp. mycoides strain Ben468; Mesoplasma florum L1; Spiroplasma culicicola

				AES−1; Ureaplasma urealyticum serovar 10 str. ATCC 33699
	Nitrospirales	Direct primary gain	-	Nitrospira moscoviensis strain NSP M−1
	Solibacteres	Direct primary gain	-	Solibacter usitatus Ellin6076
	Acidobacteriales	Direct primary gain	-	Candidatus Koribacter versatilis Ellin345; Terriglobus saanensis SP1PR4; Acidobacterium capsulatum ATCC 51196; Granulicella mallensis MP5ACT 8;
	Parachlamydiales	Direct primary gain	-	Protochlamydia naegleriophila genome assembly PNK1; Parachlamydia acanthamoebae UV−7
	Opitutae	Direct primary gain	-	Opitutus terrae PB90−1
	Deltaproteobacteria	Direct primary gain	-	Desulfovibrio africanus str. Walvis Bay; Desulfomonile tiedjei DSM 6799; Geobacter sp. M21; Geobacter uraniireducens Rf4; Myxococcus fulvus 124B02; Myxococcus stipitatus DSM 14675; Archangium gephyra strain DSM 2261; Vulgatibacter incomptus strain DSM 27710; Anaeromyxobacter dehalogenans 2CP−1; Sorangium cellulosum So0157−2; Sorangium cellulosum 'So ce 56'; Chondromyces crocatus strain Cm c5; Sandaracinus amylolyticus strain DSM 53668; Haliangium ochraceum DSM 14365
	Alphaproteobacteria	Direct primary gain	-	Gluconacetobacter diazotrophicus PAl 5;

				Tistrella mobilis KA081020-065; Porphyrobacter neustonensis strain DSM 9434; Citromicrobium sp. JL477; Erythrobacter litoralis strain DSM 8509; Altererythrobacter atlanticus strain 26DY36; Croceicoccus naphthovorans strain PQ-2; Sphingopyxis terrae NBRC 15098 strain 203-1; Blastomonas sp. RAC04; Blastomonas sp. RAC04; Novosphingobium aromaticivorans DSM 12444; Sphingomonas melonis; Sphingomonas sanxanigenens DSM 19645; Sphingobium sp. SYK−6; Caulobacteraceae bacterium OTSz_A_272; Caulobacter sp. K31; Phenylobacterium zucineum HLK1; Brevundimonas sp. GW460−12−10−14−LB 2; Asticcacaulis excentricus CB 48; Bradyrhizobium icense strain LMTR 13; Rhodopseudomonas palustris BisB5; Oligotropha carboxidovorans OM5; Nitrobacter winogradskyi Nb−255; Methylocella silvestris BL2; Beijerinckia indica subsp. indica ATCC 9039; Methylocystis sp. SC2; Starkeya novella DSM 506 anthobacter

				autotrophicus Py2; Azorhizobium caulinodans ORS 571; Rhodoplanes sp. Z2−| C6860; Chelatococcus daeguensis strain TAD1; Bosea vaviloviae strain Vaf18; Mesorhizobium loti MAFF303099; Chelativorans sp. BNC1; Martelella endophytica strain | C6887; Aminobacter aminovorans strain KCTC 2477; Hoeflea sp. IMCC20628 ; Neorhizobium galegae chromid pHAMBI540a; Agrobacterium tumefaciens strain S33; Ensifer adhaerens strain Casida A; Sinorhizobium americanum CCGM7; Ochrobactrum anthropi strain OAB; Filomicrobium sp. ; Hyphomicrobium denitrificans ATCC 51888; Rhodomicrobium vannielii ATCC 17100; Methyloceanibacter caenitepidi; Devosia sp. H5989; Pelagibacterium halotolerans B2; Rhizobium phaseoli strain R650; Shinella sp. HZN7; Parvibaculum lavamentivorans DS−1;
		-	Direct secondary loss	Alpha proteobacterium HIMB59; Novosphingobium pentaromativorans US6−1; Sphingopyxis sp. LPB0140; Zymomonas mobilis subsp. pomaceae ATCC 29192; Maricaulis maris MCS10; Micavibrio aeruginosavorus EPB; Magnetospirillum sp.

				M−1; Rhodospirillum rubrum ATCC 11170; Haematospirillum jordaniae strain H5569; Thalassospira xiamenensis M−5 = DSM 17429; Liberibacter crescens BT-1; Brucella abortus strain 63 75; Bartonella tribocorum; Magnetospira sp. QH−2
		Sequential secondary gain	-	Defluviimonas alba strain cai42; Rhodobacter sphaeroides ATCC 17025; Sulfitobacter sp. AM1-D1; Celeribacter indicus strain P73
	Gammaproteobacteria	Direct primary gain	-	Luteibacter rhizovicinus strain LJ96T; Lysobacter capsici strain 55; Lysobacter antibioticus strain ATCC 29479; Dokdonella koreensis DS−123; Dyella jiangningensis strain SBZ 3−12; Pseudoxanthomonas spadix BD−a59; Stenotrophomonas maltophilia D457; Xanthomonas gardneri strain ICMP 7383;
		-	Direct secondary loss	Xylella fastidiosa 9a5c plasmid pXF51; Xanthomonas albilineans str. GPE PC73;
	Betaproteobacteria	Direct primary gain	-	Nitrosospira briensis C−128; Methylovorus glucosetrophus SIP3-4; Massilia sp. WG5 plasmid unnamed 2
		Sequential primary gain	-	Janthinobacterium sp. 1_2014MBL_MicDiv; Pandoraea apista strain DSM 16535; Pandoraea sputorum strain DSM 21091;Collimonas

				fungivorans Ter331; Herbaspirillum seropedicae SmR1; Paraburkholderia caribensis strain Bcrs1W; Burkholderia pseudomallei Pasteur 52237; Burkholderia pseudomallei strain Burk178-Type2; Azoarcus sp. KH32C plasmid pAZKH DNA; Achromobacter xylosoxidans genome assembly NCTC10807; Bordetella flabilis strain AU10664; Bordetella holmesii ATCC 51541; Advenella kashmirensis WT001; Polyangium brachysporum strain DSM 7029; Rubrivivax gelatinosus IL144 DNA; Delftia sp. HK171; Mitsuaria sp. 7; Roseateles depolymerans strain KCTC 42856; Acidovorax ebreus TPS|; Acidovorax citrulli AAC00-1; Ramlibacter tataouinensis TTB310; Variovorax paradoxus EPS; Polaromonas sp. JS666; Hydrogenophaga sp. PBC;
		Direct tertiary gain	-	Cupriavidus basilensis strain 4G11; Ralstonia mannitolilytica strain SN82F48; Thiobacillus denitrificans ATCC 25259;
		-	Direct secondary loss	Basilea psittacipulmonis DSM 24701; Alcaligenes faecalis strain ZD02; Castellaniella; defragrans 65Phen; Bordetella pertussis strain E476; Pusillimonas sp. T7−7; Taylorella equigenitalis ATCC 35865;

	Gammaproteobacteria	Direct primary gain	-	Legionella hackeliae genome assembly LHA; Tatlockia micdadei genome assembly LMI; Coxiella burnetii CbuG_Q212; Pseudomonas syringae pv. tomato str. DC3000; Pseudomonas stutzeri DSM 4166; Halomonas chromatireducens strain AGD 8−3
		-	Direct secondary loss	Mutant Legionella pneumophila subsp. pneumophila str.; Hextuple_3a Legionella pneumophila str. Corby;
		Direct secondary gain	-	Pseudomonas aeruginosa strain PA_D16

Note: This table doesn't contain *NHEJ*- to *LigD/Ku* events that do not culminate to NHEJ+ eventually and *LigD/Ku* to *NHEJ*- events. Other organisms not listed in the table do not code for NHEJ by the virtue of the eubacterial ancestor.

Supplementary table 9

Variable	Test statistics	K value	λ value	$logL_0$	logL	P-value
Genome size	Pagel's λ	-	0.91	95.07	426.25	$4.56x10^{-146}$
Genome size	Blomberg's K	0.0144	-	-	-	$1x10^{-3}$
rRNA copy number	Pagel's λ	-	0.899	-179.85	183.493	$4.68x10^{-160}$
rRNA copy number	Blomberg's K	0.00368	-	-	-	$1x10^{-3}$

Table depicting the strength of phylogenetic signal for genome size and rRNA copy number across a phylogenetic tree of 969 bacteria using two test statistics – Pagel's λ and Blomberg's K - employed for continuous character traits. Both the measures suggest that phylogenetic conservatism is significantly greater than random expectations for both the genome characteristics, albeit there is a discordance between the strength of phylogenetic signal for both the genome characteristics measured by Pagel's λ and Blomberg's K.
